# Advanced Flow Cytometry Analysis Algorithms for Optimizing the Detection of “Different From Normal” Immunophenotypes in Acute Myeloid Blasts

**DOI:** 10.3389/fcell.2021.735518

**Published:** 2021-09-28

**Authors:** Carmen-Mariana Aanei, Richard Veyrat-Masson, Lauren Rigollet, Jérémie Stagnara, Emmanuelle Tavernier Tardy, Elisabeth Daguenet, Denis Guyotat, Lydia Campos Catafal

**Affiliations:** ^1^Laboratoire d’Hématologie, Centre Hospitalier Universitaire de Saint-Étienne, Saint-Étienne, France; ^2^Laboratoire d’Hématologie, Centre Hospitalier Universitaire de Clermont-Ferrand, Clermont-Ferrand, France; ^3^Institut de Cancérologie Lucien Neuwirth, Saint-Priest-en-Jarez, France

**Keywords:** AML with recurrent genetic abnormalities, Infinicyt, Citrus, viSNE, SPADE, different-from-normal (DfN)

## Abstract

Acute myeloid leukemias (AMLs) are a group of hematologic malignancies that are heterogeneous in their molecular and immunophenotypic profiles. Identification of the immunophenotypic differences between AML blasts and normal myeloid hematopoietic precursors (myHPCs) is a prerequisite to achieving better performance in AML measurable residual disease follow-ups. In the present study, we applied high-dimensional analysis algorithms provided by the Infinicyt 2.0 and Cytobank software to evaluate the efficacy of antibody combinations of the EuroFlow AML/myelodysplastic syndrome panel to distinguish AML blasts with recurrent genetic abnormalities (*n* = 39 AML samples) from normal CD45^low^ CD117+ myHPCs (*n* = 23 normal bone marrow samples). Two types of scores were established to evaluate the abilities of the various methods to identify the most useful parameters/markers for distinguishing between AML blasts and normal myHPCs, as well as to distinguish between different AML groups. The Infinicyt Compass database-guided analysis was found to be a more user-friendly tool than other analysis methods implemented in the Cytobank software. According to the developed scoring systems, the principal component analysis based algorithms resulted in better discrimination between AML blasts and myHPCs, as well as between blasts from different AML groups. The most informative markers for the discrimination between myHPCs and AML blasts were CD34, CD36, human leukocyte antigen-DR (HLA-DR), CD13, CD105, CD71, and SSC, which were highly rated by all evaluated analysis algorithms. The HLA-DR, CD34, CD13, CD64, CD33, CD117, CD71, CD36, CD11b, SSC, and FSC were found to be useful for the distinction between blasts from different AML groups associated with recurrent genetic abnormalities. This study identified both benefits and the drawbacks of integrating multiple high-dimensional algorithms to gain complementary insights into the flow-cytometry data.

## Introduction

The application of flow cytometry in the clinical evaluation of hematological diseases has undergone a constant evolution over time. Flow cytometric immunophenotyping is widely accepted as an indispensable method for the diagnosis and monitoring of lymphoid pathologies. In contrast, for hematological pathologies characterized by a wide heterogeneity of neoplastic cell population, such as the acute myeloid leukemias (AMLs), multicolor flow cytometry (MFC) is still considered a complementary tool in diagnosis and follow-up. Multicenter studies have reported relatively high numbers of false-positive cases when measurable residual disease (MRD) in AML was examined, resulting in a low specificity of 71%, even when using standardized protocols, likely due to differences in subjective interpretation (Brooimansa et al., 2019). The European LeukemiaNet Minimal Residual Disease Working Party has recommended the different-from-normal (DfN) approach and automated data analysis testing for optimization of MFC to establish MRD in AML (Schuurhuis et al., 2018).

The manual processing based on Boolean gating of the high-dimensional dataset obtained in AML immunophenotyping is highly subjective, time-consuming, not easily scalable to a high number of dimensions, and is inherently inaccurate because it does not account for cell population overlap (Lo et al., 2009; O’Donnell, 2013; Pedreira et al., 2019). Therefore, new algorithms for MFC data gating have been developed, including principal component analysis (PCA), which draws out the underlying variance within a dataset and is a widely used tool for visualizing multidimensional data (Matos et al., 2017). For visualization of common patterns within datasets, clustering algorithms have also been developed (Matos et al., 2017).

The automatic population separator (APS), canonical correlation analysis (CCA), neighbor-APS (NAPS) diagrams based on PCA algorithm and t-distributed stochastic neighbor embedding (t-SNE) based on K-means clustering algorithm are implemented in the Infinicyt software developed by EuroFlow (EF) to allow comparison of cell populations from different case groups with a database (Pedreira et al., 2019).

Citrus software also allows detection of differences between groups and correlate or predict experimental outcomes (Mair et al., 2016). In addition, Citrus is the only algorithm that automatically identifies statistically significant differences between groups of cases (Kimball et al., 2018).

Other algorithms for automatic gating and 2D mapping of high-dimensional datasets have been developed, such as spanning-tree progression analysis of density-normalized events (SPADE; Qiu et al., 2011) and t-SNE–based algoritm (viSNE) (Amir el et al., 2013).

The identification of algorithms useful for the unsupervised analysis of MFC data in AML is the first step in developing automated data analysis tools. This retrospective study aimed to evaluate the usefulness of novel MFC data analysis software for comparing the immunophenotypic profile of myeloid hematopoietic precursors (myHPCs) from normal bone marrow (NBM) samples with that of leukemic blasts from AML cases carrying recurrent genetic abnormalities, such as t(8;21), t(15;17), inv(16), and *MLL* (*KMT2A*) gene alterations. Data were acquired in a standardized manner and were analyzed using the Compass algorithm in the Infinicyt software and the Citrus, viSNE, and SPADE algorithms from the Cytobank software.

Here, we present some advantages and disadvantages of different unsupervised MFC analysis tools for evaluating the performance of the EF AML/myelodysplastic syndrome (MDS) antibody panel for (EF AML/MDS panel), for the identification of the most useful markers for separating AML blasts from their nearest normal myHPCs counterpart, and for the identification of the AML group-specific phenotypic imprints.

## Materials and Methods

### Study Groups

Samples from 39 AML patients, comprised of seven patients with t(8;21)(q22;q22) *RUNX1/RUNX1T1* AML, 13 patients with t(15;17)(q24;q21) *PML/RAR*α AML, eight patients with inv(16)(p13;q22) or t(16;16)(p13;q22) *CBFB/MYH11* AML, and 11 patients with *MLL/KMT2A* gene-altered AML (*MLL* AML), diagnosed at the Institut de Cancérologie Lucien Neuwirth (Saint-Priest-en-Jarez, France) between 2013 and 2017, were analyzed and compared with data from 23 NBM samples (14 healthy donors, nine patients undergoing sternotomy for cardiac surgery) from the University Hospitals of Saint-Étienne and Clermont-Ferrand, France.

Written informed consent was obtained from each patient and healthy donor, as approved by the institutional procedures of the independent Ethics Committee and the Comité de Protection des Personnes – Île-de-France (NCT03233074/17.07.2017).

The patients’ characteristics are summarized in Supplementary Table 1. The diagnosis of AML was established according to the current World Health Organization (WHO) criteria (Swerdlow et al., 2016). Healthy donors and cardiac surgery patients included in the study had normal blood counts and without any evidence of a hematopoietic disease.

### Flow Cytometry

Bone marrow aspirates were collected on K2-EDTA anticoagulant and 3 × 10^6^ cells (10^6^ per tube) were stained with the first three EF AML/MDS panel antibody combinations (van Dongen et al., 2012), which allow evaluation of the three main bone marrow (BM) myeloid lineages: neutrophilic (Tube 1), monocytic (Tube 2), and erythroid (Tube 3) (see Supplementary Table 2). Sample preparation and acquisition were performed according to the EF standard operating procedure (Kalina et al., 2012). In order to avoid that the measurements may be hampered by the background fluorescence of antigen-negative cells and other particles (i.e., debris) the cytometer setup [including photomultiplier tube (PMT) voltage gains and compensation] was performed according EF instrument settings procedure (Kalina et al., 2012). Appropriate instrument performance and laboratory procedures were confirmed by the results obtained in the FranceFlow and EF quality assessment rounds (Kalina et al., 2015; Solly et al., 2019).

At least 500,000 total events were acquired by tube using three FACS Canto II cytometers (Becton–Dickinson, San Jose, CA, United States) in two university-affiliated hospitals in France (Saint-Étienne and Clermont-Ferrand). Data were analyzed using Infinicyt 2.0 software (Cytognos, Salamanca, Spain) and the software packages including the algorithms Citrus, viSNE, and SPADE from the Cytobank (Cytobank, Santa Clara, CA, United States; downloaded from http://cytobank.org).

### Flow Cytometry Data Analysis

Pre-gating for intact singlets followed by discrimination of normal myHPCs and AML blasts was performed in Infinicyt 2.0 software primarily using the backbone markers CD117, human leukocyte antigen-DR (HLA-DR), and CD45 (Supplementary Figures 1C, 2C, 3C, 4C) in line with EuroFlow recommandations (van Dongen et al., 2012). Several exclusion gates were used to avoid inclusion of undesirable events that may fall into the “blast gate,” such as CD11b+ hypogranular neutrophils and basophils (Tube 1), CD14^low^ granulocytes and CD14+ CD300e (IREM-2)+ monocytes (Tube 2), and CD10+ hematogones (Tube 1) (Supplementary Figures 1C,D, 2C, 3C, 4C).

Using this strategy, the initial bulk of myHPCs included the very immature CD117+ CD34+ precursors and the more mature CD117+ CD34− myHPCs from each of the three myeloid lineages.

Regarding AML cases, this strategy allowed discrimination of both immature blasts (CD34+ CD117+), and those more matures (CD34− CD117+); the percentages of the blasts identified by MFC based on this strategy corresponded to those identified in morphology (see Supplementary Table 1).

Subsequently, events corresponding to myHPCs and AML blasts were exported to new FCS files and analyzed using the Compass tool from the Infinicyt 2.0 and in Citrus, viSNE, and SPADE from Cytobank software. The ArcSinh-hyperbolic transformed data using a co-factor of 150 were subjected to analysis with the three listed algorithms from Cytobank for the following markers (FSC-A, SSC-A, CD45, CD34, HLA-DR, CD117, CD16, CD13, CD10, and CD11b for the neutrophilic lineage, Tube 1; FSC-A, SSC-A, CD45, CD34, HLA-DR, CD117, CD64, CD35, CD300e, and CD14 for the monocytic lineage, Tube 2; and FSC-A, SSC-A, CD45, CD34, HLA-DR, CD117, CD36, CD33, CD105, and CD71 for the erythroid lineage, Tube 3). In Infinicyt software, the negative visibility is by default corrected when the Logical scales are used.

The all markers from the tubes, including the three markers of the backbone used for the pre-gating of AML blasts and myHPCs, have been used in analysis in order to identify potential differences in their intensity of expression.

### Construction of Infinicyt Databases

In the Infinicyt software, a database was built for each of the three myeloid lineages: neutrophilic (Tube 1 database), monocytic (Tube 2 database), and erythroid (Tube 3 database). The final databases for neutrophil, monocytic, and erythroid lineages contained cases belonging to five groups: NBM (normal myHPCs), t(8;21) AML, t(15;17) AML, inv(16) AML, and *MLL* AML.

In accordance with EuroFlow recommendations (Kalina et al., 2012), the files were verified for the appropriate staining of backbone markers, which are essential for appropriate gating of the cells of interest.

Four FCS files from samples stained with Tube 1 and Tube 2 antibody combinations and three FCS files for Tube 3 were excluded from the databases for the NBM group. These files showed >2.5 SD discordances compared with the group median for the markers used to select myHPCs and for the side scatter (SSC) values of the internal control populations, lymphocytes, neutrophils, and NRCs (Supplementary Figure 1A).

In the AML files, the inadequate staining of markers used for AML blast gating, such as CD117 and HLA-DR, was also observed for the control populations (lymphocytes and NRCs; >2.5 SD of the group mean). Therefore, one FCS file was excluded from the neutrophil lineage database for the t(8;21) AML group, one FCS file was excluded from all databases for the t(15;17) AML group, and one FCS file was excluded from the neutrophil and monocytic lineage databases for the *MLL* AML group (Supplementary Figures 2A,B, 3A,B, 4A,B).

Each group of cases was subsequently plotted in a balanced principal component analysis (PCA) plot with medians for each patient and SD curves for each group of cases to compare each case with each of the five groups and for each of three myeloid lineages. The markers that received a weight over 10% in the first or second principal component in an APS view of the AML blasts and the nearest normal myHPCs were considered useful for distinguishing between groups (Figures 2–5A). These results have been compared with data showed by Parameter Band Histograms (Supplementary Figure 5).

For the configuration of Cytobank analysis software (Citrus, viSNE, SPADE) and data interpretation, we used the practical guide published by Kimball et al. (2018).

### Citrus Algorithm Settings

The FCS files containing the manually gated myHPCs and AML blasts were imported into Cytobank and then subjected to Citrus analysis using the following settings: (1) singlet, non-debris cell as input; (2) hierarchical clustering in order to group cells into populations; (3) analysis of 10 out of 12 possible parameters per tube (antibodies outlined in Supplementary Table 2 and mentioned in the previous section “Flow Cytometry Data Analysis”); (4) files assigned to appropriate experimental groups [e.g., AML t(8;21) vs. NBM]; (5) nearest shrunken centroid (R package PAMR)^1^ association model; (6) cluster characterization of medians; (7) equal event sampling; (8) 5,000 events sampled per file; (9) 5% minimum cluster size (Cytobank default); (10) five-fold cross-validation (Cytobank default); and (11) 1% false discovery rate (Cytobank default).

### Spanning-Tree Progression Analysis of Density-Normalized Events Algorithm Settings

Manually gated singlet events corresponding to myHPCs in NBM and for the AML blasts were imported into Cytobank and then subjected to SPADE analysis using the following settings: (1) target number of nodes = 20; and (2) 100% percent downsampling. Analysis was performed using 10 of 12 possible parameters per tube (as mentioned in the previous section “Flow Cytometry Data Analysis”), and no fold change calculations were made for this data set.

### Cellular Abundance Evaluation Using the R SPADE Package

Spanning-tree progression analysis of density-normalized events trees were initially visually investigated to identify nodes that were potentially different in cellular abundance between conditions. Two SPADE runs of datasets were performed. Clusters organization by analyzed group (AML or NBM) were not conserved between runs, resulting in considerable differences between the SPADE tree from the two runs (Supplementary Figure 6), and rendering SPADE inappropriate for this analysis.

### viSNE Algorithm Settings

Manually gated singlet events corresponding to myHPCs in NBM and to AML blasts were imported into Cytobank^2^ and then subjected to viSNE analysis.

According to previously published data (Byford et al., 2018; Kimball et al., 2018; Gonder et al., 2020), viSNE was used as a non-linear technique to reduce dimensionality, but also to identify the phenotypically related cell cluster as assigned by the parameters tSNE1 and tSNE2.

viSNE clustering analysis was performed on 10 of 12 possible parameters (as mentioned in the previous section “Flow Cytometry Data Analysis”). Equal event sampling was selected at 100,000 events per individual (the lowest common denominator across all samples). The advanced settings were set as per the Cytobank default settings: random seed, 1,000 iterations, perplexity = 30, theta = 0.5.

Data accompanying each viSNE node were downloaded from Cytobank to calculate the frequency of events in each node, followed by testing for statistical significance.

### Characterization and Comparison of Normal Myeloid Hematopoietic Precursors and Acute Myeloid Leukemia Blasts Using Spanning-Tree Progression Analysis of Density-Normalized Events and viSNE Algorithms From Cytobank

**FIGURE 1:**
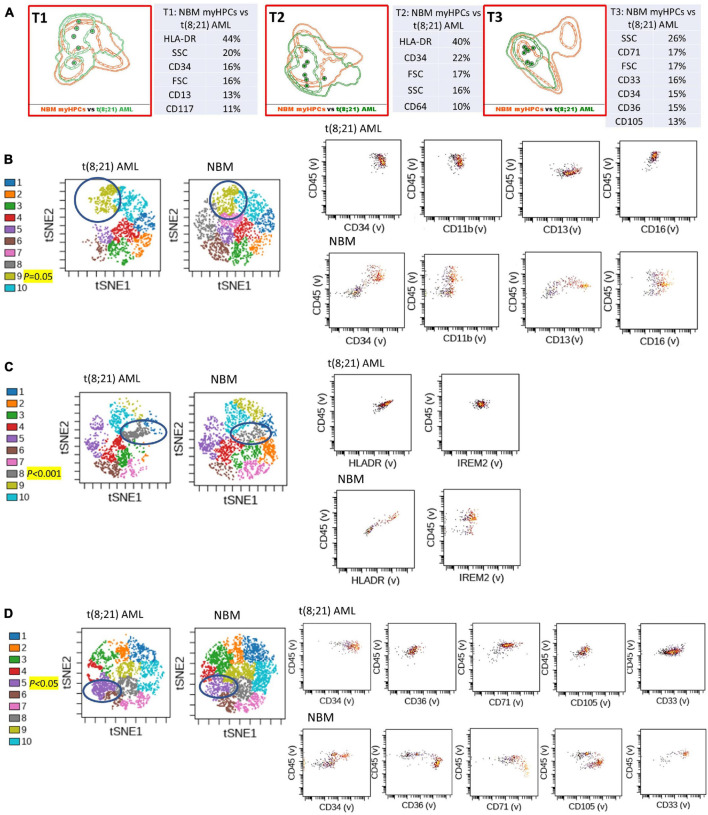
Phenotypic differences between t(8;21) AML blasts and the normal myHPCs using Tubes 1–3 of the EF AML/MDS panel and analysis with viSNE algorithms or Infinicyt analysis. **(A)** Tube 1 (T1) of the EuroFlow (EF) acute myeloid leukemia (AML)/myelodysplastic syndrome (MDS) panel. Tube 2 (T2), EF AML/MDS panel. Tube 3 (T3), EF AML/MDS panel. Automated population separator (APS) views of illustrating principal component analyses (PCA; using Infinicyt software) of t(8;21) AML (green) and normal myeloid hematopoietic precursor (myHPCs, orange) samples, based on the expression of the eight markers included in the T1, and FSC, SSC parameters (left panel), T2 (middle panel), and T3 (right panel). Tube 1: 6 t(8;21) AML, 19 normal myHPCs; Tube 2: 7 t(8;21) AML, 19 normal myHPCs; Tube 3: 7 t(8;21) AML, 21 normal myHPCs. Dots represent the median values of individual cases, the dotted line represents the 1 SD curve of the group and the solid line represents the 2 SD curve. The table shows the contribution of each parameter to the first (PC1, *x*-axis) or second (PC2, *y*-axis) principal component reflected as percent values. **(B–D left panel)** Representative viSNE diagrams showing the nodes with a significant difference between t(8;21) AML and normal bone marrow (NBM) cases (node 9 T1, node 8 T2, and node 5 T3 are indicated by blue circles on the plot, and highlighted in yellow with significance level). **(B–D right panel)** Dot plots also show significant differences in the expression of markers on myHPCs from NBM versus t(8;21) AML blasts.

**FIGURE 2:**
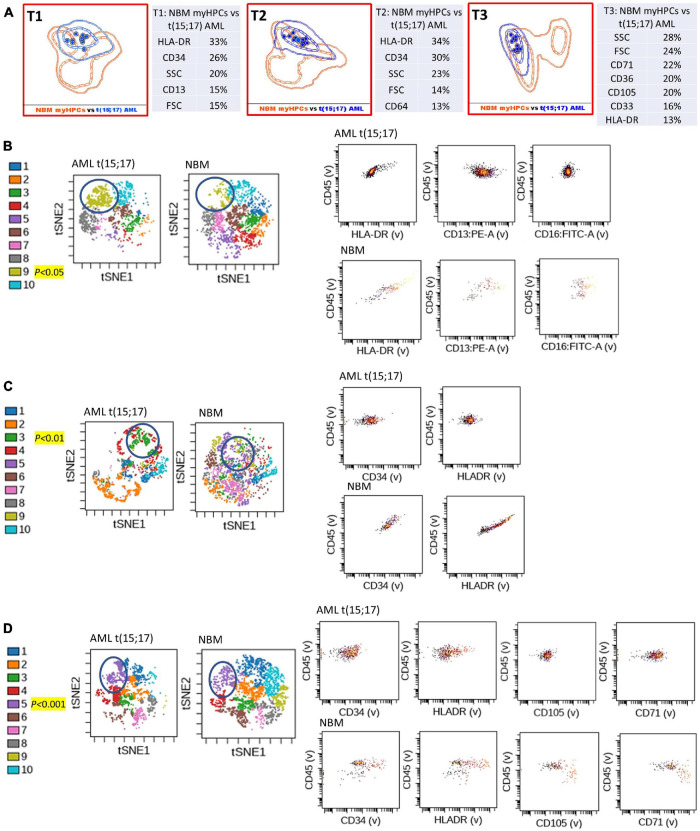
Phenotypic differences between t(15;17) AML blasts and the normal myHPCs using Tubes 1–3 of the EF AML/MDS panel and analysis with viSNE algorithms or Infinicyt analysis. **(A)** Tube 1 (T1) of the EuroFlow (EF) acute myeloid leukemia (AML)/myelodysplastic syndrome (MDS) panel. Tube 2 (T2), EF AML/MDS panel. Tube 3 (T3), EF AML/MDS panel. Automated population separator (APS) views of illustrating principal component analyses (PCA; using Infinicyt software) of t(15;17) AML (blue) and normal myeloid hematopoietic precursor (myHPCs, orange) samples, based on the expression of the eight markers included in the T1, and FSC, SSC parameters (left panel), T2 (middle panel), and T3 (right panel). Tube 1: 12 t(15;17) AML, 19 normal myHPCs; Tube 2: 12 t(15;17) AML, 19 normal myHPCs; Tube 3: 12 t(15;17) AML, 21 normal myHPCs. Dots represent the median values of individual cases, the dotted line represents the 1 SD curve of the group and the solid line represents the 2 SD curve. The table shows the contribution of each parameter to the first (PC1, *x*-axis) or second (PC2, *y*-axis) principal component reflected as percent values. **(B–D left panel)** Representative viSNE diagrams showing the nodes with a significant difference between t(15;17) AML and normal bone marrow (NBM) cases (node 9 T1, node 3 T2, and node 5 T3 are indicated by blue circles on the plot, and highlighted in yellow with significance level). **(B–D right panel)** Dot plots also show significant differences in the expression of markers on myHPCs from NBM versus t(15;17) AML blasts.

**FIGURE 3:**
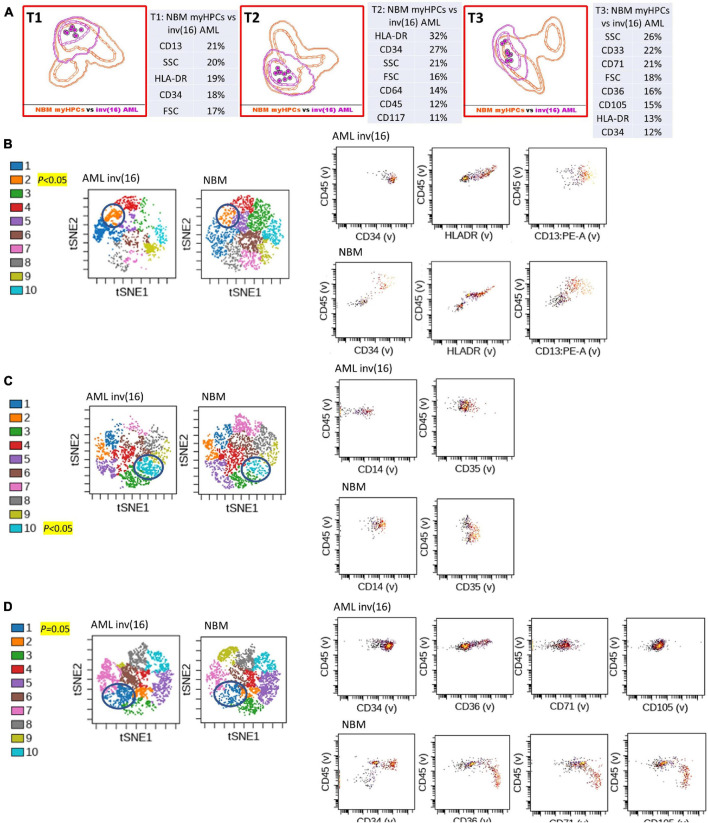
Phenotypic differences between inv(16) AML blasts and the normal myHPCs using tubes 1–3 of the EF AML/MDS panel and analysis with viSNE algorithms or Infinicyt analysis. **(A)** Tube 1 (T1) of the EuroFlow (EF) acute myeloid leukemia (AML)/myelodysplastic syndrome (MDS) panel. Tube 2 (T2), EF AML/MDS panel. Tube 3 (T3), EF AML/MDS panel. Automated population separator (APS) views of illustrating principal component analyses (PCA; using Infinicyt software) of inv(16) AML (violet) and normal myeloid hematopoietic precursor (myHPCs, orange) samples, based on the expression of the eight markers included in the T1, and FSC, SSC parameters (left panel), T2 (middle panel), and T3 (right panel). Tube 1: 8 inv(16) AML, 19 normal myHPCs; Tube 2: 8 inv(16) AML, 19 normal myHPCs; Tube 3: 8 inv(16) AML, 21 normal myHPCs. Dots represent the median values of individual cases, the dotted line represents the 1 SD curve of the group and the solid line represents the 2 SD curve. The table shows the contribution of each parameter to the first (PC1, *x*-axis) or second (PC2, *y*-axis) principal component reflected as percent values. **(B–D left panel)** Representative viSNE diagrams showing the nodes with a significant difference between inv(16) AML and normal bone marrow (NBM) cases (node 2 T1, node 10 T2, and node 1 T3 are indicated by blue circles on the plot, and highlighted in yellow with significance level). **(B–D right panel)** Dot plots also show significant differences in the expression of markers on myHPCs from NBM versus inv(16) AML blasts.

**FIGURE 4:**
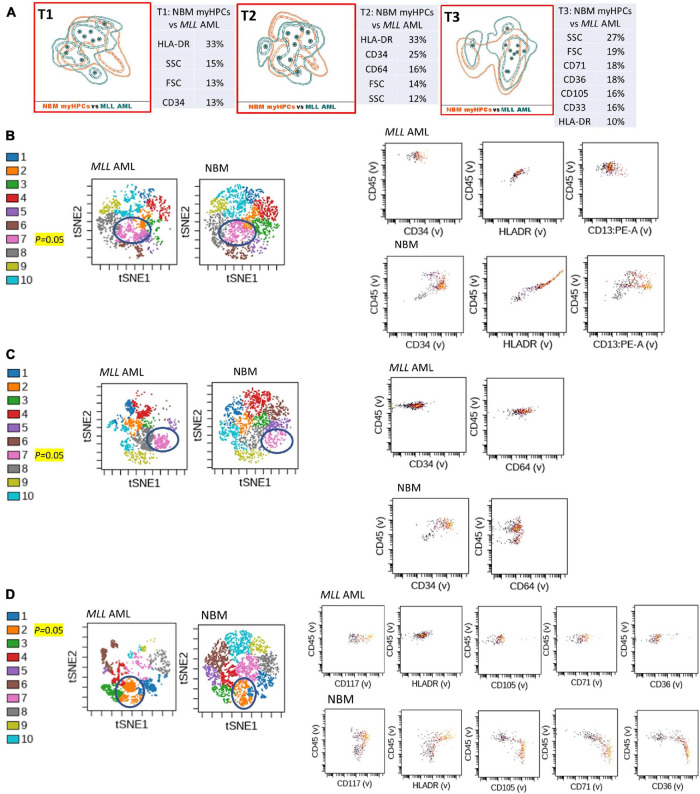
Phenotypic differences between *MLL* AML blasts and the normal myHPCs using Tubes 1–3 of the EF AML/MDS panel and analysis with viSNE algorithms or Infinicyt analysis. **(A)** Tube 1 (T1) of the EuroFlow (EF) acute myeloid leukemia (AML)/myelodysplastic syndrome (MDS) panel. Tube 2 (T2), EF AML/MDS panel. Tube 3 (T3), EF AML/MDS panel. Automated population separator (APS) views of illustrating principal component analyses (PCA; using Infinicyt software) of *MLL* AML (turquoise) and normal myeloid hematopoietic precursor (myHPCs, orange) samples, based on the expression of the eight markers included in the T1, and FSC, SSC parameters (left panel), T2 (middle panel), and T3 (right panel). Tube 1: 10 *MLL* AML, 19 normal myHPCs; Tube 2: 10 *MLL* AML, 19 normal myHPCs; Tube 3: 11 *MLL* AML, 21 normal myHPCs. Dots represent the median values of individual cases, the dotted line represents the 1 SD curve of the group and the solid line represents the 2 SD curve. The table shows the contribution of each parameter to the first (PC1, *x*-axis) or second (PC2, *y*-axis) principal component reflected as percent values. **(B–D left panel)** Representative viSNE diagrams showing the nodes with a significant difference between *MLL* AML and normal bone marrow (NBM) cases (node 7 T1, node 7 T2, and node 2 T3 are indicated by blue circles on the plot, and highlighted in yellow with significance level). **(B–D right panel)** Dot plots also show significant differences in the expression of markers on myHPCs from NBM versus *MLL* AML blasts.

The nodes that showed the most statistically significant differences in the cellular abundance between groups (indicated by blue circles; Figures 1–4) were then evaluated in viSNE: the relative expression of the markers was evaluated in dot plots and the intensity of the expression of markers in heat maps (Supplementary Figures 7–10). The multiple comparative tests were performed to identify significant differences in markers expression between the different study groups.

### Statistical Analysis

Statistical comparisons were performed using a two-tailed *t*-test. The fcs data were arcsine transformed before analysis, therefore we assume that they follow a Gaussian distribution.

The distribution of variables was measured using one-tailed paired and unpaired *t*-tests for single comparisons and one-way analysis of variance (ANOVA) and Kruskal–Wallis tests for multiple comparisons.

The identification of significant nodes in the cellular abundance between groups was determined using Microsoft Excel 2019 (Microsoft Office, Las Vegas, NV, United States). The Kruskal–Wallis multiple comparative test from GraphPad Prism 5^TM^ (GraphPad Software, San Diego, CA, United States) was used to compare the differences in markers expression between AML blasts from different groups and myHPCs as reflected by viSNE algorithms.

### Scoring System for Identification of the Most Informative Markers to Distinguish Between Acute Myeloid Leukemia Blasts and Normal Hematopoietic Progenitor Cells and to Evaluate the Analysis Algorithms

A scoring system was developed for identification of the most useful markers for discrimination between the AML blasts and the normal myHPCs and for testing the capacity of different methods for their identification (Table 1). The assignment of the score weight was based on measurement of the intensity of expression of the antigen-positive cells by flow cytometry. Internal negative controls to set the positive/negative threshold have been used to reliably distinguish between a positive and a negative population of cells in accordance with previously published recommendations (Hulspas et al., 2009). For each backbone and additional markers from EF AML/MDS panel a score between 0 and 2 was attributed to denote differences between AML blasts and the normal MyHPCs as was reflected by each method.

**TABLE 1:** Scoring system for identification of the most informative markers to distinguish between AML blasts and normal HPCs and to evaluate the analysis algorithms.

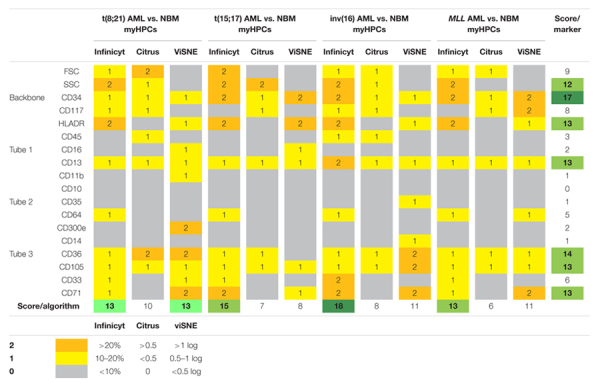

*Orange = score 2, yellow = score 1, dark green, green, and light green represent the highest values obtained for the sum of the scores (in descending order). The total score to evaluate the discriminative capacity of each marker to distinguish between AML blasts and normal myHPCs was obtained by adding all values obtained by the respective marker or parameter for distinguishing between each AML group and normal HPCs and for each analysis method. The threshold score for marker relevance was >10. The bold values highlight the highest values obtained for the sum of the scores.*

In Infinicyt software, the differences between AML blasts and normal hematopoietic progenitor cells (HPCs) were visualized in separate, non-fixed APS plots using all eight markers and the FSC and SSC parameters of each tube in line with previously published studies (Costa et al., 2010; Flores-Montero et al., 2016; Theunissen et al., 2017). The relative contribution that each marker or parameter made to distinguish between the blasts of different AML groups and normal myHPCs was scored based on weights of over 20% in the first or second principal component in an Automatic Population Separator (APS) (2 points), between 10 and 20% (1 point), or under 10% (0 points).

For the other software, the differences in the intensity of markers on the populations of interest was assessed on the scales of the plots provided by the different software, after the arcsine transformation of the data, which allow a normal distribution of the data and improves the representation of cells with minimal fluorescence, as it was demonstrated by Herzenberg et al. (2006).

Citrus boxplots were used to evaluate differences between the analyzed markers and parameters. Differences in markers expression and FSC, SSC between AML blasts and normal HPCs > 0.5 between AML blasts and normal HPCs received 2 points, those inferior to 0.5 received 1 point and the absence of difference was noted with 0 points.

Logarithmic differences were used to interpret markers and parameter differences with viSNE. Differences in markers expression and FSC, SSC between AML blasts and normal HPCs > 1 log received 2 points; those between 0.5 and 1 log, 1 point and the absence of difference was noted with 0 points.

The total score to evaluate the discriminative capacity of each marker to distinguish between AML blasts and normal myHPCs was obtained by adding all values obtained by the respective marker or parameter for distinguish between each AML group and normal HPCs and for each analysis method. The threshold score for marker relevance was >10.

The total score used to evaluate the effectiveness of each algorithm for the ability to distinguish AML blasts from normal myHPCs was obtained by summing the values obtained of each two-by-two paired group [t(8;21) AML versus normal myHPCs, t(15;17) AML versus normal my HPCs, inv(16) AML versus normal myHPCs, and *MLL* AML versus normal myHPCs].

### Scoring System to Evaluate the Most Informative Markers/Parameters to Distinguish the Blasts From Different Acute Myeloid Leukemia Groups With Tubes 1–3 of the EuroFlow Acute Myeloid Leukemia/Myelodysplastic Syndrome Panel When Using the Principal Component Analysis-Based Analysis

The separation between different AML groups was scored based on an overlap of the 2 SD curves: no overlap between 2 SD curves (2 points); overlap of the 2 SD and the 1 SD curve: 1 point; overlap of both 1 SD curves: 0 points.

**FIGURE 5:**
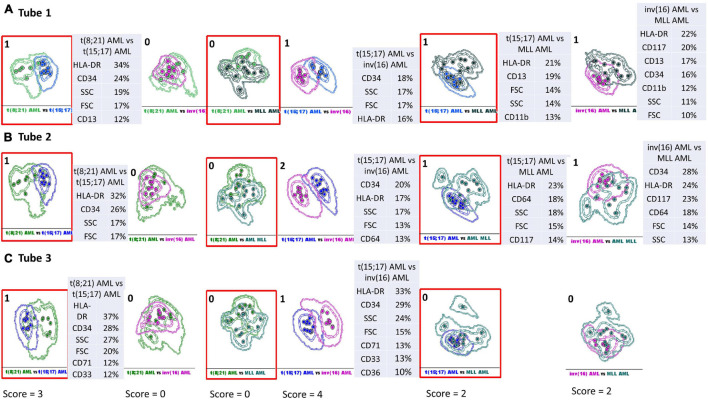
Infinicyt database-guided analyses aid identification of phenotypic differences between AML groups with different genetic abnormalities. Samples were stained with antibodies from Tube 1–3 of the EuroFlow (EF) acute myeloid leukemia (AML)/myelodysplastic syndrome (MDS) panel. Sample groups: t(8;21) acute myeloid leukemia (green), t(15;17) AML (blue), inv(16) AML (violet), *MLL* AML (turquoise). Dots represent the median values of individual cases; the dotted line represents the 1 SD curve of the group and the solid line represents the 2 SD curve. The tables show the contribution of each parameter to the first (PC1, *x*-axis) or second (PC2, *y*-axis) principal component reflected as percent values. The separation between different AML groups was scored based on an overlap of the 2 SD curves: no overlap between 2 SD curves (2 points); overlap of the 2 SD and the 1 SD curve: 1 point; overlap of both 1 SD curves: 0 points. The total score to evaluate the discriminative capacity between the AML blasts from different AML groups was obtained by summing the values obtained in tubes 1–3 EF AML/MDS for each two-by-two paired group [t(8;21) AML versus t(15;17) AML, t(8;21) AML versus inv(16) AML, t(8;21) AML versus MLL AML, t(15;17) AML versus inv(16) AML, t(15;17) AML versus MLL AML, and inv(16) AML versus MLL AML]. **(A)** Phenotypic differences between AML groups with different genetic abnormalities observed with Tube 1 EF AML/MDS panel. **(B)** Phenotypic differences between AML groups with different genetic abnormalities observed with Tube 2 EF AML/MDS panel. **(C)** Phenotypic differences between AML groups with different genetic abnormalities observed with Tube 3 EF AML/MDS panel.

The total score to evaluate the discriminative capacity between the AML blasts from different AML groups was obtained by summing the values obtained in tubes 1–3 EF AML/MDS for each two-by-two paired group [t(8;21) AML versus t(15;17) AML, t(8;21) AML versus inv(16) AML, t(8;21) AML versus *MLL* AML, t(15;17) AML versus inv(16) AML, t(15;17) AML versus *MLL* AML, and inv(16) AML versus *MLL* AML] (Figure 5).

## Results

We sought to evaluate whether the selection of myHPCs and AML blasts using primarily the backbone markers present in Tubes 1–3 of EF AML/MDS panel would allow appropriate gating and discrimination between normal myHPCs and AML blasts, as well as between blasts from different groups of AML cases.

The most common subtypes of AML with recurrent genetic abnormalities – AML t(8;21), AML t(15;17), AML inv(16), and *MLL* AML – were used in this study on assumption that they might constitute homogeneous groups in terms of immunophenotype.

We used two clustering algorithms, Citrus and SPADE, and two dimensionality-reduction methods, viSNE, and a PCA-based algorithm (Compass, Infinicyt) to explore the immunophenotypic features associated with AML blasts compared with those associated with normal myHPCs.

### Citrus, Spanning-Tree Progression Analysis of Density-Normalized Events, viSNE, and Compass-Based Analyses Do Not Allow for Clear Distinctions to Be Made Between Acute Myeloid Leukemia Blasts and Normal Myeloid Hematopoietic Precursors When Selected Primarily by Backbone Markers but Can Improve the Identification of Phenotypic Differences

**FIGURE 6:**
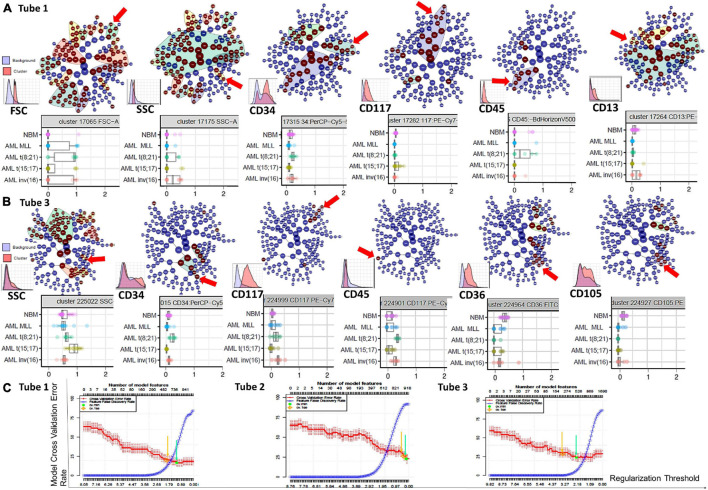
Phenotypic differences identified by the Citrus algorithms between AML blasts and normal myHPCs using Tubes 1–3 of the EF AML/MDS panel. Citrus-defined radial hierarchical plots **(A)** colored by FSC, SSC, CD34, CD117, CD45, and CD13 expression (Tube 1 of the EF AML/MDS panel), and **(B)** colored by SSC, CD34, CD117, CD45, CD36, and CD105 expression (Tube 3 of the EF AML/MDS panel). CITRUS trees in which each node denotes different cell clusters. The red arrow illustrate cell clusters were the median marker intensities differed statistically significantly between the five groups [NBM, *MLL* AML, t(8;21) AML, t(15;17) AML, and inv(16) AML] as determined by PAMR analysis (R implementation of Prediction Analysis for Microarrays). No clusters were identified in the Citrus trees when using the markers form the Tube 2 of the EF AML/MDS panel and the software default settings. Boxplots below Citrus-defined radial hierarchical plots reveals the differences of parameter expression among the different groups of cases. Box plots shows the median marker intensities for the various markers [expressing interquartile range (IQR) and median values] and values for each individual within the groups of cases. The nodes chosen to be shown in boxplots represent the parent clusters (the “highest in the hierarchy” significant node). CITRUS histograms for the parent clusters are illustrated on the top of each marker. For each marker, the histogram shows the marker expression on the cells of interest in the specific cluster (red) against the marker expression on all other cells (blue). **(C)** Citrus-generated model error rate plots. Cv_1se was used with a 5 fold cross validation and a false discovery rate of 0.01 was applied. Each plot displays the cross-validation (CV) error rate (red line) and the false discovery rate (blue line) for the CV constrained models corresponding to each tube (from left to right: Tubes 1 to 3 of EF AML/MDS panel). Vertical arrows indicate the features of two predictive CV-constrained models with varying stringency: 1 standard error CV (cv_1se), yellow arrow; minimum CV (cv.min), green arrow.

Generalized reduction in markers expression heterogeneity was observed on the cells of the nodes containing a significantly increased number of events in AML cases compared to normal myHPCs when viSNE algorithms were used, as reflected in dot plots (Figures 1–4B–D). However, no clear separation was observed between the AML blasts and normal myHPCs with any of these algorithms when using the chosen gating strategy (Figures 6A,B, 1–4A–D).

In line with previously published studies (Kotecha et al., 2010; Abel et al., 2018) we used CITRUS algorithms because allow an automated discovery of statistically significant differences between phenotypic profiles of different groups of cases. By unsupervised hierarchical clustering, cell clusters are identified, phenotypes of the cells are characterized, and differences between groups are determined. The association model PAMR with cv_1se was used to defines cross validation rate and feature false discovery rate. The error rate models chosen by the Citrus software showed that the minimum prediction error reflecting the percentage of misclassified cases was around 20% for the neutrophil lineage database (Tube 1), around 40% for the monocytic lineage database (Tube 2), and around 25% for the erythroid lineage database (Tube 3) (Figure 6C).

However, these algorithms can help to identify which are the most informative markers in distinguishing AML blasts from their nearest normal myHPCs counterparts. This step is useful for improving the antibody combinations and the gating strategy for myHPCs or AML blasts.

### Principal Component Analysis and Citrus Algorithms Indicate That Side Scatter Parameters Contribute to Distinguishing Between Acute Myeloid Leukemia Blasts and Normal Myeloid Hematopoietic Precursors

Based on the PCA analysis (Infinicyt software) and the analysis of clusters with the minimum prediction error (Citrus software), the FSC and SSC parameters may be useful for distinguishing between AML blasts and normal myHPCs. SSC seemed to be more useful for this purpose, and both algorithms highlighted the SSC increase in t(15;17) AML blasts (Figures 2A, 6B), although differences were observed for the other AML groups as well.

Although included in the analysis, the viSNE algorithm did not indicate that FSC and SSC parameters could be useful for separating AML blasts and normal myHPCs (see heatmaps Supplementary Figures 7–10).

### CD34 and HLA-DR Were Useful Backbone Markers for the Distinction Between Acute Myeloid Leukemia Blasts and Normal Myeloid Hematopoietic Precursors

Citrus boxplots corresponding to the parent nodes from the hierarchy (Figures 6A,B), the parameter band histograms from Infinicyt (Supplementary Figure 5), and the viSNE dot plots (Figures 1B,D, 2C,D, 3B,D, 4B,C) indicated that the CD34 expression was increased in t(8;21) AML and inv(16) AML, but decreased in t(15;17) AML and *MLL* AML. When comparing the CD34 MFI data of AML blasts with the normal myHPCs on the viSNE nodes containing a significantly increased number of events, we noticed a statistical significant increase in t(8;21) AML blasts vs. myHPCs (*P* = 0.0196) and in inv(16) AML blasts vs. myHPCs (*P* = 0.0205), but a significant decrease in t(15;17) AML blasts vs. myHPCs (*P* = 0.0152) and in *MLL* AML blasts vs. myHPCs (*P* = 0.0102).

The power of discrimination of CD34 seemed to be potentiated by an association with other antibodies, nearly always resulting in much better discrimination in two of the three tubes (Tubes 1 and 2), as reflected by the Compass algorithm (Infinicyt) (Figures 1–4A). Absence or decrease of the discrimination capacity of this marker between the different groups of cases in the case of Tube 3 could not have been due to other factors such as the order of addition of antibodies during cell staining because an initial premix of the backbone markers was made, thereafter was distributed in all tubes and only then were the cells added. All other steps during cell staining were performed in the same time and in identical conditions.

HLA-DR was another discriminating parameter among the backbone markers, as reflected by Infinicyt and viSNE diagrams. Similar to CD34, low expression of HLA-DR was detected on t(15;17) AML (Supplementary Figures 5, 3B–D) and *MLL* AML blasts (Figures 4B,D), with increased expression on t(8;21) AML (Supplementary Figures 5, 2C) and inv(16) AML blasts (Supplementary Figures 5, 4B). When comparing the HLA-DR MFI of AML blasts with the normal myHPCs on the viSNE nodes containing a significantly increased number of events, we noticed a statistical significant increase in t(8;21) AML blasts vs. myHPCs (*P* = 0.0439) and in inv(16) AML blasts vs. myHPCs (*P* = 0.0405), and a significant decrease in t(15;17) AML blasts vs. myHPCs (*P* = 0.0352), but not in MLL AML blasts vs. myHPCs (*P* = 0.0697). A possible explanation that no statistical significance was obtained for the intensity of expression of HLA-DR between MLL AML blasts and normal myHPCs is likely related to the important heterogeneity of expression of this marker between the different MLL AML cases, as reflected by Infinicyt histograms (Supplementary Figure 5).

The intensity of expression of CD117 contributed little to the ability to discriminate between AML blasts and normal myHPCs. According to the Citrus algorithms, CD117 was slightly increased on the t(8;21) AML, inv(16) AML blasts and slightly decreased on the t(15;17) AML, *MLL* AML blasts compared with normal HPCs with the combination of markers from the third tube of EF AML/MDS panel (Figure 6B). This observation was confirmed in the Infinicyt histograms [for t(8;21) AML, inv(16) AML blasts in all three tubes of EF AML/MDS panel (Supplementary Figures 5A,C)].

In addition, the Infinicyt histograms revealed that CD117 could be increased also in some cases of *MLL* AML (Supplementary Figure 5D) and by the viSNE (for *MLL* AML) (Figure 4D). However, CD117 expression did not significantly increase on *MLL* AML blasts from the viSNE nodes containing a significantly increased number of events compared to the normal myHPCs (*P* = 0.1990).

CD45 was the least discriminant of the backbone markers and was slightly more intensely expressed by the t(8;21) AML and inv(16) AML blasts compared to normal myHPCs, as highlighted especially by Citrus algorithms in Tube 3 (Figures 6A,B).

### All Algorithms Used Showed That CD13 Could Contribute to Discrimination Between Acute Myeloid Leukemia Blasts and Normal Myeloid Hematopoietic Precursors Among the Additional Markers in Tube 1

The tables showing the contribution of each parameter to the first (PC1, *x*-axis) or second (PC2, *y*-axis) principal component generated by the Infinicyt algorithms (Table T1 from Figures 1A, 2A, 3A) showed that CD13 contributes to the discrimination between inv(16) AML, t(15;17) AML and t(8;21) AML blasts and the normal my HPCs. The largest differences compared to normal myHPCs were recorded for this marker in the inv(16) AML group (higher intensity of expression) as revealed by the viSNE dot plots on the nodes containing a significantly increased number of events in inv(16) AML (Figure 3B; *P* = 0.0217), but also between *MLL* AML blasts (decreased expression) compared with their nearest normal myHPCs (Figure 4B; *P* = 0.0429).

Concerning other neutrophil-related markers evaluated in Tube 1, viSNE dot plots showed that CD16 was weakly discriminant between t(8;21) AML and t(15;17) AML blasts (decreased expression) and normal myHPCs (Figures 1B, 2B), and CD11b between t(8;21) AML blasts (increased expression) and normal myHPCs (Figure 1B), but these differences did not reach statistical significance. These slight differences for CD16 and CD11b reflected by viSNE dot plots were not seen using other algorithms.

### CD64 Was Slightly More Intensely Expressed by t(15;17) Acute Myeloid Leukemia and *MLL* AML Blasts Than by Normal Myeloid Hematopoietic Precursors

Compared with the performance of Tubes 1 and 3, the combination of antibodies used in Tube 2 was less able to discriminate between AML blasts and normal myHPCs. Among the markers included in this tube, the Compass algorithm (Infinicyt) showed that CD64 was somewhat discriminant, identified as being slightly more intensely expressed by t(15;17) AML and *MLL* AML blasts than by normal myHPCs (Supplementary Figure 5).

According to analysis with the viSNE algorithm, the CD300e (IREM2) marker showed increased expression on t(8;21) AML blasts, and the CD14 and CD35 markers showed decreased expression on the leukemic blasts from the AML inv(16) group, compared to the normal myHPCs in the nodes with statistically significant increased numbers of events in AML cases. However, the parameter band histogram from Infinicyt did not show a notable difference for CD300e on t(8;21) AML blasts, nor for CD14 on inv(16) AML blasts compared to normal myHPCs (Supplementary Figures 5A,C).

### CD36, CD105, and CD71 Were Discriminating Markers Between Acute Myeloid Leukemia Blasts and Normal Myeloid Hematopoietic Precursors

The CD36, CD105, and CD71 from Tube 3 were ranked by Compass-based analysis (Infinicyt) among the discriminating markers between AML blasts and normal myHPCs (Figures 1–4A). viSNE algorithms showed also that these markers were able to discriminate between AML blasts and normal myHPCs (Figures 1–4D). However, a statistically significant model from Citrus confirmed that only two of these markers, CD36 and CD105, were helpful in distinguishing the two groups (Figure 6B).

Overall, the erythroid lineage-associated antigens CD36, CD105, and CD71 have a reduced expression on the AML blasts compared to normal myHPCs (Figures 1–5D, 6B and Supplementary Figure 5 row T3). Although the PCA analysis (Infinicyt software) indicated a contribution of CD33 to discriminating between AML blasts and normal myHPCs, the parameter band histograms and viSNE algorithms supported this only for t(8;21) AML blasts (Supplementary Figure 5A and Figure 1D). This difference is most likely due to the number of parameters considered by each type of algorithm used to distinguish between the groups.

### Compass-Based Analysis (Infinicyt) Was the Best Tool for the Identification of Those Markers or Parameters With the Best Potential to Distinguish Between Acute Myeloid Leukemia Blasts and Normal Myeloid Hematopoietic Precursors

The relative contribution that each marker or parameter to distinguish between the blasts of different AML groups and normal myHPCs was scored based on weights in the first or second principal component in an APS diagram in Compass (Infinicyt), on Citrus boxplots (Cytobank) and based on logarithmic differences observed for median fluorescence intensity (MFI) with viSNE (Cytobank). The total score to evaluate the discriminative capacity of each marker to distinguish between AML blasts and normal myHPCs was obtained by adding all values on each line. The threshold score for marker relevance was >10. Compass-guided analysis showed that the best separation capacity was achieved between inv(16) AML blasts and normal myHPCs (score = 18), thereafter the separation between by t(15;17) AML blasts and normal myHPCs (score = 15), followed by the separation between t(8;21) AML and MLL AML blasts and normal myHPCs (score = 13).

The total score used to evaluate the effectiveness of each algorithm for the ability to distinguish AML blasts from normal myHPCs was obtained by summing the columns values.

Based on this score (Table 1), the Compass-based analysis (Infinicyt) was found to identify most of the differences in marker expression between AML blasts from different groups and their nearest normal myHPCs counterpart, receiving the highest rating compared with the other analyzed algorithms (59 points were assigned to Compass compared with 31 points for Citrus and 43 points for viSNE).

As revealed by all analysis algorithms, in descending order, the following seven parameters were the most useful to distinguish between AML blasts and normal myHPCs: CD34; CD36; then with equal weight HLA-DR, CD13, CD105, and CD71; and finally SSC (Table 1).

### Compass Database (Infinicyt)-Guided Analysis Allows for the Identification of Phenotypic Differences Between Acute Myeloid Leukemia Groups With Different Genetic Abnormalities

We sought to evaluate the performance of the Infinicyt software and the antibody combinations from the first three tubes of the EF AML/MDS panel for the discrimination of the blasts from different AML groups. To this end, we used a new score that evaluates the APS capacity to separate the blasts from different AML groups and to rank the different parameters and markers according to their contribution to discrimination. Details of the score calculation are presented in Figure 5. Relatively good discrimination was obtained between the leukemic cells from t(15;17) AML and the other AML groups [in descending order of degree of separation: inv(16) AML, t(8;21) AML, and *MLL* AML], and weak discrimination between inv(16) AML and *MLL* AML blasts with the markers from the Tubes 1 and 2. The most useful markers in separating the blasts from t(15;17) AML and inv(16) AML groups were CD34, HLA-DR, and the SSC parameter, followed by FSC and the markers CD64, CD71, CD36, and CD33 (Figure 5). The best-ranked markers that allowed separation between t(15;17) AML blasts and those from t(8;21) AML group were HLA-DR, CD34, SSC, FSC, followed by CD13, CD33, and CD71 (Figure 5). HLA-DR and SSC were also useful to distinguish between the t(15;17) AML blasts and those from the *MLL* AML group, followed by FSC, CD11b, CD13, CD64, and CD117 (Figure 5).

HLA-DR, CD34, and CD117 also contributed to the separation of leukemic cells from inv(16) AML from those of the *MLL* AML group, followed by CD64, CD13, and CD11b markers and SSC, FSC parameters (Figure 5).

Database-guided analysis in the Infinicyt software allowed a rapid analysis of the phenotypic differences between the blasts from different AML groups.

## Discussion

The immunophenotypic characterization of AML blasts by MFC is standard of care in AML diagnosis and follow-up. However, there is no broad consensus on the panel of antibodies to be used or on the algorithms of analysis that would allow for the standardization of practices at the international level.

The EuroFlow Consortium, a European collaborative research group has developed and validated a eight-color EF AML/MDS panel that allows the detection of AML blasts, their lineage assignment, and the accurate evaluation of the maturation profiles of myeloid lineages (van Dongen et al., 2012; Matarraz et al., 2017; Orfao et al., 2019). However, the discriminatory potential between myHPCs and AML blasts using this panel or its utility for AML blast classification in different cytogenetic groups is not known.

This preliminary, retrospective study was performed to evaluate the different tools provided by different software for data analysis to evaluate whether the first three tubes of the EF AML/MDS panel could be used for discrimination between myHPCs and AML blasts using this panel or its utility for AML blast classification in different cytogenetic groups.

The different methods analysis had a range of advantages and disadvantages.

The major advantage of the Citrus algorithm (Cytobank) is the capacity to build predictive models that allows identification of the minimum set of biomarkers necessary to discriminate effectively between the groups and estimation of the accuracy with which these biomarkers can do this (Polikowsky and Drake, 2019).

Of the others Cytobank algorithms, the SPADE analysis provides information about the population structure and the heterogeneity of population events, and the viSNE tool aids with the identification of distinct phenotypes between the groups of cases.

However, the software on the Cytobank platform is less practical for multicenter studies because it does not allow overlapping data with different nomenclature for antibodies or fluorochromes which is frequently observed in these types of studies.

The SPADE software allows the creation of population trees reflecting the size of cell clusters and the relationships between them. In agreement with other published data, a major drawback of the SPADE interface is the loss of single-cell resolution (Chester and Maecker, 2015), but also the considerable differences observed between the SPADE tree from the different runs when applied SPADE to the same datasets (Amir el et al., 2013). In our opinion, another major drawback of SPADE is the inability to directly perform statistical analyses on the frequency of events; instead, data must be exported to external software to perform these operations, which can be a time-consuming process.

The viSNE software from Cytobank has also a disadvantage; the configuration of this software is operator-dependent. In particular, the operator sets the number of iterations, which specifies the number of runs of the algorithm, and the perplexity parameter, which influences the results.

Another limitation of the viSNE tool was the difficulty we encountered when attempting to compare groups of cases, for example in visualization of differences of markers’ expression using heatmaps. When the number of parameters to be analyzed is great or the number of cases to compare is increased, the heatmap visualization and interpretation are difficult.

Infinicyt 2.0 also allows the evaluation of the relationship between different cell clusters from a sample, or between different groups based on two new diagrams, the robust curve and population burst (Pedreira et al., 2019). However, the distinct benefit of this software is offered by the PCA-based diagrams, which allows automatic n-dimensional separation of the events in clusters based on the expression of all markers used for cell staining.

In Infinicyt 2.0, several new diagrams have been recently developed, such as t-SNE, Canonical Correlation Analysis (CA), Neighborhood Automatic Population Separator (NAPS), and Compass VI (CVI 1) diagrams, that also allow exploration of population heterogeneity. These are not expert-based decisions, and are therefore not associated with a significant component of individual subjectivity (Pedreira et al., 2019).

The major advantage of the Infinicyt software is the possibility to perform the database-guided analysis. Compass-guided method is a rapid analysis tool, allowing comparison of the data acquired across different flow cytometry platforms, which was specially designed for a better visualization when comparing groups of cases. This method gives information about the parameters that are useful for separation of the groups, and therefore, can help to improve the combinations of antibodies. The Compass database-guided analysis based on PCA algorithm has proven its advantage in various studies, such as for acute leukemia orientation (Lhermitte et al., 2018), B cell chronic lymphoproliferative disorders classification (Costa et al., 2010), multiple myeloma diagnosis and monitoring (Flores-Montero et al., 2016), and B-cell acute lymphoblastic leukemia follow-up (Theunissen et al., 2017).

Our preliminary data show that the Compass database-guided analysis is a helpful tool for the identification of DfN patterns that may advance actual methods of minimal residual disease evaluation by MFC in AML, which are frequently based on the detection of leukemia-associated immunophenotypes (LAIPs) identified at diagnosis.

Based on EF experience, the unequivocal identification of blast cells in BM samples is possible using solely the backbone markers from the EF AML/MDS panel (van Dongen et al., 2012). The use of a backbone consisting of four combined markers can be a good strategy if the differences in the prevalence of individual antigen expression in AML blasts are taken into consideration. The prevalence of marker expression in AML blasts [after the exclusion of t(15;17) AML blasts] was 76.5% for CD34, 79.78% for CD117, and 90.12% for HLA-DR, as reported by Webber et al. (2008).

However, our data revealed that a strategy based solely on the expression of these markers cannot be used to clearly isolate the AML blasts with recurrent genetic abnormalities or normal myHPCs, due to the inability to avoid the contamination of the CD45^low^ CD117+ CD34± HLA-DR^variable (low to high)^ population with undesirable mature cells.

Sandes et al. (2013) showed that the evaluation of blasts by MFC using the “CD34+ or CD117+ HLA-DR+” phenotypic profile cannot replace morphological blast count evaluation in MDS; instead, the combined use of both evaluation methods may provide complementary information, increasing the accuracy and reproducibility of bone marrow blast cell counts in these patients.

Therefore, evaluating the capability of other markers for the distinction of AML blasts from normal myHPCs counterparts may be useful.

CD38 appears to be a very useful marker, as the 92% of AML blasts express this protein, as reported by Webber et al. (2008).

The data from our current study showed that the inclusion of myeloid lineage-associated markers, such as CD13, CD64, CD36, CD105, CD71, and CD33, in the gating strategy can increase the discriminatory power for distinguishing between AML blasts and normal HPCs that are committed toward different myeloid lineages.

In line with this observation, Webber et al., noticed that second to CD38 expression, AML blasts most frequently expressed the myeloid lineage markers CD13 (91%) and CD33 (87%) (Webber et al., 2008).

The CD64 is an useful marker for the distinction of early monocytic precursors, whereas CD105, CD36, and CD71 are useful for the identification of myeloid precursors that have committed to the erythroid lineage (Kalina et al., 2012).

The Compass database-guided AML analysis (Infinicyt) is a user-friendly interface, facilitating the rapid evaluation of differences between blasts from different types of AML. The identification of phenotypic imprints associated with AML groups with recurrent genetic abnormalities may allow for the detection of residual leukemic blasts, even in the absence of phenotypic identification at diagnosis.

In our study, except for the t(15;17) AML group, PCA did not result in the clear distinction between AML blasts from varying groups, primarily due to the heterogeneity of marker expression across blasts harboring the same recurrent genetic abnormality. For example, the t(8;21) AML group displayed large variability in the expression of CD34, HLA-DR, and CD13, whereas, for *MLL* AML blasts, the most important differences were observed for CD34, CD117, HLA-DR, CD13, CD11b, CD64, CD36, CD105, CD71, and CD33, in addition to the FSC and SSC parameters (Supplementary Figure 5).

The reduced number of cases included in the t(8;21) AML and *MLL* AML groups represents the primary limitation of this study because these small sample sizes did not allow for the identification of possible subtypes inside these immunophenotypic heterogenous groups.

However, this preliminary study has allowed re-designing a 12-color panel combining backbone markers from the EF AML/MDS panel with the myeloid lineage-associated markers, such as CD13, CD64, CD36, CD105, CD71, and CD33 that could improve the discriminatory potential between myHPCs and AML blasts. This 12-color panel should be initially tested on homogenous phenotypic groups of AMLs (as is expected in the case of AML with recurrent genetic abnormalities) or AMLs having a well-established molecular target to be able to compare MFC data with molecular biology results in different time points of AML follow-up. Multicenter studies using the Compass database-guided analysis based on PCA algorithm is required for its validation. To this end, the following steps should be followed: the use of standardized protocols for sample preparation, and data acquisition, effective harmonization of the instruments between different centers, intra-center normalization of the instrument(s) using the daily 8 Peak Beads QC, correction of the compensations for the data files, correction of the mean fluorescence intensities of the cell surface markers to adjust the median values and eliminate the antibody batches variations between instruments [i.e., using a Python script, as previously described by Le Lann et al. (2020)], together with robust expertise in data analysis.

Differences observed in the results obtained with these two types of software, Infinicyt and Cytobank maybe are related to the different development directions of the two packages. Cytognos strive efforts in the clinical classification functions, whereas Cytobank is focused on development of multidimensional analysis tools like tSNE and SPADE.

The PCA-based algorithms can be efficiently used to analyze data with low dimensionality, but heterogenous. However, the results obtained depends on the discriminatory capacity of the antibody combinations used.

In addition, high-dimensional analysis algorithms may help visualizing the phenotypic changes of the AML blasts under the pressure of microenvironmental factors and to explore the immune system disorders contributing to the AML onset and progression. Recent studies bring to front also the role of the interaction between bone, immune and cancer cells in protecting the latter from immune system attack (Gnoni et al., 2020). Data generated in this type of studies requires the design of robust data analysis algorithms to overcome the difficulties raised by big data volumes, heterogeneous data distributions, and complex and dynamic data characteristics, and these new tools of MFC data analysis may contribute to the definition of the role of these cells in the of disease pathogenesis and to the identification of the new therapeutic targets.

In conclusion, the new tools of data analysis in MFC: (1) avoid data misinterpretations; (2) allow study of well-defined population groups; (3) highlight useful markers for better discrimination between different clinical groups; and (4) enhance our understanding of the utility of the panels used in routine practice.

The benefits of using these analysis tools are becoming increasingly evident, as evidenced by the growing number of studies that use them.

## Data Availability Statement

The datasets presented in this study can be found in online repositories. The names of the repository/repositories and accession number(s) can be found below: https://flowrepository.org/, FR-FCM-Z3JL.

## Ethics Statement

The studies involving human participants were reviewed and approved by the Comité de Protection des Personnes – Île-de-France (NCT03233074/17.07.2017). The patients/participants provided their written informed consent to participate in this study.

## Author Contributions

C-MA: conceptualization, formal analysis, data curation, and writing – original draft preparation. C-MA, RV-M, LR, and JS: validation. ET and DG: clinical investigation. C-MA, RV-M, LR, JS, ET, LC, ED, and DG: writing – review and editing. All authors have read and agreed to the published version of the manuscript.

## Conflict of Interest

The authors declare that the research was conducted in the absence of any commercial or financial relationships that could be construed as a potential conflict of interest.

## Publisher’s Note

All claims expressed in this article are solely those of the authors and do not necessarily represent those of their affiliated organizations, or those of the publisher, the editors and the reviewers. Any product that may be evaluated in this article, or claim that may be made by its manufacturer, is not guaranteed or endorsed by the publisher.

## Footnotes

1
https://cran.r-project.org/web/packages/pamr

2
www.cytobank.org

## Supplementary Material

Supplementary Figure 1 Study design and the strategy of analysis for the identification of normal myHPCs in NBM samples using the Tubes 1–3 of EF AML/MDS panel. **(A)** Schematic overview of the normal bone marrow (NBM) files included in the databases. FCS files corresponding to the healthy donor (HD) and cardiac surgery (CS) bone marrow (BM) samples stained with antibodies from the EuroFlow (EF) acute myeloid leukemia (AML)/myelodysplastic syndrome (MDS) panel Tube 1 (T1), Tube 2 (T2), and Tube 3 (T3). **(B)** Multidimensional (2 SD principal component-based) views from Tubes 1 to 3 showing homogenous staining for the backbone markers on the internal control populations (lymphocytes, Ly; neutrophils; and nucleated red cells, NRC) from the NBM files included in the databases. **(C)** Analysis strategy for HPC selection in Tubes 1, 2, and 3 of the EF AML/MDS panel. **(D)** Mature cell populations that may contaminate the blast’s gate when the gating strategy is based only on backbone markers. T1, Tube 1; T2, Tube 2.

Supplementary Figure 2 Study design and the strategy of analysis for the identification of AML blasts using Tube 1 of the EF AML/MDS panel. **(A)** Schematic overview showing the AML files included in the Tube 1 AML database. **(B)** Multidimensional (2 SD principal component-based) views showing homogenous staining for the backbone markers on the internal control populations (lymphocytes, Ly; nucleated red cells, NRC) from the AML FCS files included in the Tube 1 AML database. **(C)** Analysis strategy for selection of AML blast in Tube 1 of the EF AML/MDS panel: t(8;21) AML blasts, yellow-green; t(15;17) AML blasts, light blue; inv(16) AML blasts, violet; *MLL* AML blasts, turquoise; neutrophils, pink; NRC, dark red; lymphocytes, green.

Supplementary Figure 3 Study design and the strategy of analysis for the identification of AML blasts using Tube 2 of the EF AML/MDS panel. **(A)** Schematic overview showing the AML files included in the Tube 2 AML database. **(B)** Multidimensional (2 SD principal component-based) views showing homogenous staining for the backbone markers on the internal control populations (lymphocytes, Ly; nucleated red cells, NRC) from the AML FCS files included in the Tube 2 AML databases. **(C)** Analysis strategy for selection of AML blast in Tube 2 of the EF AML/MDS panel: t(8;21) AML blasts, yellow-green; t(15;17) AML blasts, light blue; inv(16) AML blasts, violet; t(9;11) AML blasts, turquoise; neutrophils, pink; NRC, dark red; lymphocytes, green.

Supplementary Figure 4 Study design and the strategy of analysis for the identification of AML blasts using Tube 3 of the EF AML/MDS panel. **(A)** Schematic overview showing the AML files included in the Tube 3 AML database. **(B)** Multidimensional (2 SD principal component-based) views showing homogenous staining for the backbone markers on the internal control populations (lymphocytes, Ly; nucleated red cells, NRC) from the AML FCS files included in the Tube 3 AML databases. **(C)** Analysis strategy for selection of AML blast in the Tube 3 of the EF AML/MDS panel: t(8;21) AML blasts, yellow-green; t(15;17) AML blasts, light blue; inv(16) AML blasts, violet; t(9;11) AML blasts, turquoise; neutrophils, pink; NRC, dark red; lymphocytes, green.

Supplementary Figure 5 Parameter Band Histograms showing markers expression on AML blasts versus normal myHPCs. Columns display the phenotype of acute myeloid leukemia (AML) blasts: **(A)** t(8;21) AML blasts, green; **(B)** t(15;17) AML blasts, blue; **(C)** inv(16) AML blasts, violet; **(D)** *MLL* AML blasts, turquoise. Normal myeloid hematopoietic precursor (myHPC) reference images: orange discontinuous line. T1–3 rows correspond to each of Tubes 1–3 of the EuroFlow (EF) AML/myelodysplastic syndrome (MDS) panel. Histograms show the relative expression of each analyzed marker on different groups of AML blasts; circles represent the mean of an individual case.

Supplementary Figure 6 Two SPADE runs of pre-gated AML blasts stained with antibody combinations from Tube 1 EF AML/MDS panel from five t(8;21) AML cases were visually investigated to identify nodes that were potentially different in cellular abundance compared with myHPCs from NBM samples. In these plots each node comprises a group of phenotypically related cells. The size and the color of the nodes indicate the abundance and the expression, respectively. Each row represents one of the two SPADE runs and each column represents the SPADE tree obtained for one t(8;21) AML case. Clusters group by AML case are not conserved between runs, resulting in considerable differences between the SPADE tree from the two runs.

Supplementary Figure 7 viSNE cluster heatmaps colored by expression level of indicated markers for AML blasts from a representative t(8;21) AML case (top panel) compared with myHPCs from a NBM sample (bottom panel). Green boxes highlights the nodes containing significantly more events in t(8;21) AML case compared with NBM setting. **(A)** Tube 1 panel EF AML/MDS. **(B)** Tube 2 panel EF AML/MDS. **(C)** Tube 3 panel EF AML/MDS.

Supplementary Figure 8 viSNE cluster heatmaps colored by expression level of indicated markers for AML blasts from a representative t(15;17) AML case (top panel) compared with myHPCs from a NBM sample (bottom panel). Green boxes highlights the nodes containing significantly more events in t(15;17) AML case compared with NBM setting. **(A)** Tube 1 panel EF AML/MDS. **(B)** Tube 2 panel EF AML/MDS. **(C)** Tube 3 panel EF AML/MDS.

Supplementary Figure 9 viSNE cluster heatmaps colored by expression level of indicated markers for AML blasts from a representative inv(16) AML case (top panel) compared with myHPCs from a NBM sample (bottom panel). Violet boxes highlights the nodes containing significantly more events in inv(16) AML case compared with NBM setting. **(A)** Tube 1 panel EF AML/MDS. **(B)** Tube 2 panel EF AML/MDS. **(C)** Tube 3 panel EF AML/MDS.

Supplementary Figure 10 viSNE cluster heatmaps colored by expression level of indicated markers for AML blasts from a representative MLL AML case (top panel) compared with myHPCs from a NBM sample (bottom panel). Turquoise boxes highlights the nodes containing significantly more events in MLL AML case compared with NBM setting. **(A)** Tube 1 panel EF AML/MDS. **(B)** Tube 2 panel EF AML/MDS. **(C)** Tube 3 panel EF AML/MDS.

Supplementary Table 1 Patients’ characteristics.

Supplementary Table 2 Antibody combination used for evaluation of the three main BM myeloid lineages.
