# Supplementary material for: Advanced Flow Cytometry Analysis Algorithms for Optimizing the Detection of “Different From Normal” Immunophenotypes in Acute Myeloid Blasts
Source: Front Cell Dev Biol. 2021 Sep 28;9:735518. doi: 10.3389/fcell.2021.735518 (PMC8506133; doi:10.3389/fcell.2021.735518)
Supplement: Supplementary Figure 1 — Study design and the strategy of analysis for the identification of normal myHPCs in NBM samples using the Tubes 1–3 of EF AML/MDS panel. (A) Schematic overview of the normal bone marrow (NBM) files included in the databases. FCS files corresponding to the healthy donor (HD) and cardiac surgery (CS) bone marrow (BM) samples stained with antibodies from the EuroFlow (EF) acute myeloid leukemia (AML)/myelodysplastic syndrome (MDS) panel Tube 1 (T1), Tube 2 (T2), and Tube 3 (T3). (B) Multidimensional (2 SD principal component-based) views from Tubes 1 to 3 showing homogenous staining for the backbone markers on the internal control populations (lymphocytes, Ly; neutrophils; and nucleated red cells, NRC) from the NBM files included in the databases. (C) Analysis strategy for HPC selection in Tubes 1, 2, and 3 of the EF AML/MDS panel. (D) Mature cell populations that may contaminate the blast’s gate when the gating strategy is based only on backbone markers. T1, Tube 1; T2, Tube 2. [file Data_Sheet_1.PDF]

Table S1. Patients' characteristics

					Morphology examination		% of blasts by MFC
Gender	Age (Y)	Karyotype	Morphologic Diagnosis	WBC ($x10^9/L$)	PB Blasts	BM Blasts	BM Blasts/ Mean of 3 tubes
F	47	46,XX,t(15;17)(q24;q21)[15]/46,XX[5]	AML M3	1.15	1 blast/smear	26%*	55.40%
M	49	47,XY,+8[3]/46,XY[17].ish ins(15;17)(q24;q12q12)[8]	AML M3	1.69	30.0%	41.0%	46.50%
M	42	46,XY,t(15;17)(q24;q21)[20]	AML M3	12.64	72.4%	62.5%	58.70%
M	68	46,XY,t(15;17)(q24;q21)[10]/46,XY[1]	AML M3	15.38	57.7%	91.5%	84.90%
M	67	47,XY,+8,t(15;17)(q24;q21)[20]	AML M3	1.01	0.5%	68.5%	71.10%
F	29	46,XX,t(15;17)(q24;q21)[2]/44,XX,der(1)t(1;17;?)(p11;?;?),del(3)(q11),der(12)t(1;12)(?;p13),der(15)t(15;17),-17,-18[12]/46,XX[6]	AML M3	1.19	0.0%	33%*	54.90%
M	22	46,XY,t(15;17)(q24;q21)[17]	AML M3v	41.85	95.0%	94.0%	80.90%
M	35	46,XY,t(15;17)(q24;q21)[15]	AML M3	3.73	40.1%	74.5%	71.90%
M	63	46,XY,t(15;17)(q24;q21)[11]/47,sl,+8[5]/46,sl,der(11)t(11;12)(p15;q13),del(12)(q13)[2]/46,XY[2]	AML M3	4.94	78.6%	90.0%	82.40%
F	55	46,XX,t(15;17)(q24;q21)[20]	AML M3	6.27	69.8%	65.0%	67.20%
F	66	46,XY,t(15;17)(q24;q21)[15]	AML M3v	40.73	81.5%	91.5%	91.80%
F	41	46,XX,t(15;17)(q24;q21)[10]/46,XX,del(7)(p15p13),der(15)t(15;17),ider(17)(q10)t(15;17)[10]	AML M3	5.51	47.2%	48.0%	43%
M	41	46,XY,t(15;17)(q24;q21)[10]/47,idem,+8[8]/46,XY[2]	AML M3	2.14	25.0%	77.0%	71.10%
M	35	46,XY,t(8;16;21)(q22;q24;q22)[1]/45,idem,-Y[18]/46,XY[1]	AML M1	2.18	24.4%	44.0%	23.5%*
M	12	45,X,-Y,t(8;21)(q22;q22)[20]	AML?	13.07	16.4%	12.5%*	10.3%*
F	37	46,XX,t(8;21)(q22;q22)[17]/46,XX[2]	AML (M4/5?)	2.46	25.5%	66.5%	54%
M	63	46,XY,t(8;21)(q22;q22)[2]/47,idem,+13[8]	AML	192.33	96.7%	99.0%	92.20%
F	55	46,XX,t(8;21)(q22;q22),del(11)(q21;q24)[13]/45,idem,-X[4]/46,idem,del(9)(q21q31)[3]	AML M2	11.8	53.0%	62.5%	40%*
M	71	45,X,-X,t(8;21)(q22;q22)[9]/45,idem,der(2)t(2;8)(q32;q13)[11]	AML M4?	7.51	18.6%	20.5%	18.80%
F	54	46,XX,t(8;21)(q22;q22)[20]	AML M1	30.92	85.0%	87.5%	82%
F	35	46,XX,inv(16)(p13q22)[17]	AML M4 Eo variant	6.68	20.3%	22.0%	29.60%
F	20	46,XX,inv(16)(p13q22)[17]	AML M4	307.98	70.4%	87.5%	76.80%
F	30	46,XX,inv(16)(p13q22)[17]	AML M3v	16.86	45.5%	64.5%	65.30%
F	67	46,XX,inv(16)(p13q22)[17]	AML M5	65.45	29.0%	70.0%	68.60%
M	1	46,XY,inv(16)(p13q22)[7]/idem,t(1;2;4)(q22;p13;q22)[9]/idem,t(1;2;4)(q22;p13;q22),del(6)(q?)[5]	AML M4 Eo variant	147.92	60.4%	62.0%	59%
M	36	47,XY,+8,inv(16)(p13q22)[18]/46,XY[2]	AML M5	3.84	5.3%	25.5%	19.90%
M	40	46,XX,inv(16)(p13q22)[17]	AML M5	308.76	85.6%	84.0%	75.30%
M	62	46,XY,inv(16)(p13q22)[20]	AML M4 Eo variant	43.93	62.5%	72.5%	77.50%
M	67	49,XY,+8,t(9;11)(p22;q23),del(15)(q14q21),+19,+21[18]/46,XY[2]	AML M5a	3.88	49.0%	79.5%	72.50%
M	50	46,XY,der(3)(pter->3q23::11q23->11q23::10p12->10pter),der(10)(3qter->3q23 ::11q21->11q23 ::10p12->10qter),der(11)ins(10 ;11)(p12 ;q23q21)[15]:	AML M5a	10.7	31.0%	85.5%	82.60%
F	46	46,XX,t(6;11)(q27;q23),der(11)t(11;17;16)(p14;q2?;?p1?2),der(19)t(17;19)(q?2;p13)[24]/46,XX[2]	AML M1	38.47	83.4%	87.0%	88%
M	78	46,XY,t(9;11)(p22;q23)[15]/46,XY[5]	AML	6.06	0.0%	72.0%	73.65%
M	50	46,XY,t(6;11)(q27;q23)[19]/46,XY[1]	AML M5a	183.71	NA	85.5%	74.80%
M	57	46,XY,t(6;11)(q25-27;q23)[20]	AML	59.3	68.0%	90.0%	74%*
M	58	46,XX,t(9;11)(p21;q23),t(9;22)(q34;q11)[20]	AML	68.42	90.5%	95.5%	92%
M	78	47,XY,del(5)(q13q33),+8,der(11)t(11;11)(p1?3;q2?1),+der(11)t(11;11)(p1?3;q2?1),-17[cp6]	AML	3.75	3.0%	22.0%	22.80%
F	70	78~82,XX,+1,+1,+2,+4,+der(5)?t(5;12)(q31;p13)x2,+del(6)(p22)x2,+del(7)(q22),+8,+9,+9,+10,+10,+11,+11,+der(12)?t(5;12)x2,+13,+13,+14,+14,+15,+17,+17,-18,+19,+21,+21,+4~8mar[20]	AML M1	48.02	77.3%	91.0%	73%*
F	73	46,XX,der(4)t(4;11)(p16;q22)[7]/46,XX[13]	AML M1	109.88	67.5%	92.0%	88.60%
M	68	47,XY,+11[20]/46,XY[4]	AML M4	7.35	21.0%	30.0%	35%

Y, years; WBC, white blood cells; PB, peripheral blood; BM, bone marrow;
* indicate hemodiluted bone marrow aspirates

Table S2.
Antibody combination used for evaluation of the three main BM myeloid lineages

Tube	FITC	PE	PerCPCy5.5	PECy7	APC	APC-H7	Pacific Blue	V500
1	**CD16**<br>Clone CLB Fc gran/1, 5D2<br>Sanquin<br>20 µL	**CD13**<br>Clone L138<br>BD Biosciences<br>7 µL	**CD34**<br>Clone 8G12<br>BD Biosciences<br>10 µL	**CD117**<br>Clone 104D2D1<br>Beckman Coulter<br>5 µL	**CD11b**<br>Clone D12<br>BD Biosciences<br>5 µL	**CD10**<br>Clone HI10A<br>BD Biosciences<br>5 µL	**HLA-DR**<br>Clone L243<br>BioLegend<br>1 µL (dil 1:5)	**CD45**<br>Clone 2D1<br>BD Biosciences<br>1 µL
2	**CD35**<br>CloneE11<br>BD Biosciences<br>5 µL	**CD64**<br>Clone 10.1<br>BD Biosciences<br>20 µL	**CD34**<br>Clone 8G12<br>BD Biosciences<br>10 µL	**CD117**<br>Clone 104D2D1<br>Beckman Coulter<br>5 µL	**CD300e**<br>Clone UP-H2<br>Immunostep<br>5 µL	**CD14**<br>Clone MøP9<br>BD Biosciences<br>5 µL	**HLA-DR**<br>Clone L243<br>BioLegend<br>1 µL (dil 1:5)	**CD45**<br>Clone 2D1<br>BD Biosciences<br>1 µL
3	**CD36**<br>Clone CLB-IVC7<br>Sanquin<br>5 µL	**CD105**<br>Clone 266<br>BD Biosciences<br>5 µL	**CD34**<br>Clone 8G12<br>BD Biosciences<br>10 µL	**CD117**<br>Clone 104D2D1<br>Beckman Coulter<br>5 µL	**CD33**<br>Clone P67.6<br>BD Biosciences<br>10 µL	**CD71**<br>Clone M-A712<br>BD Biosciences<br>2 µL	**HLA-DR**<br>Clone L243<br>BioLegend<br>1 µL (dil 1:5)	**CD45**<br>Clone 2D1<br>BD Biosciences<br>1 µL

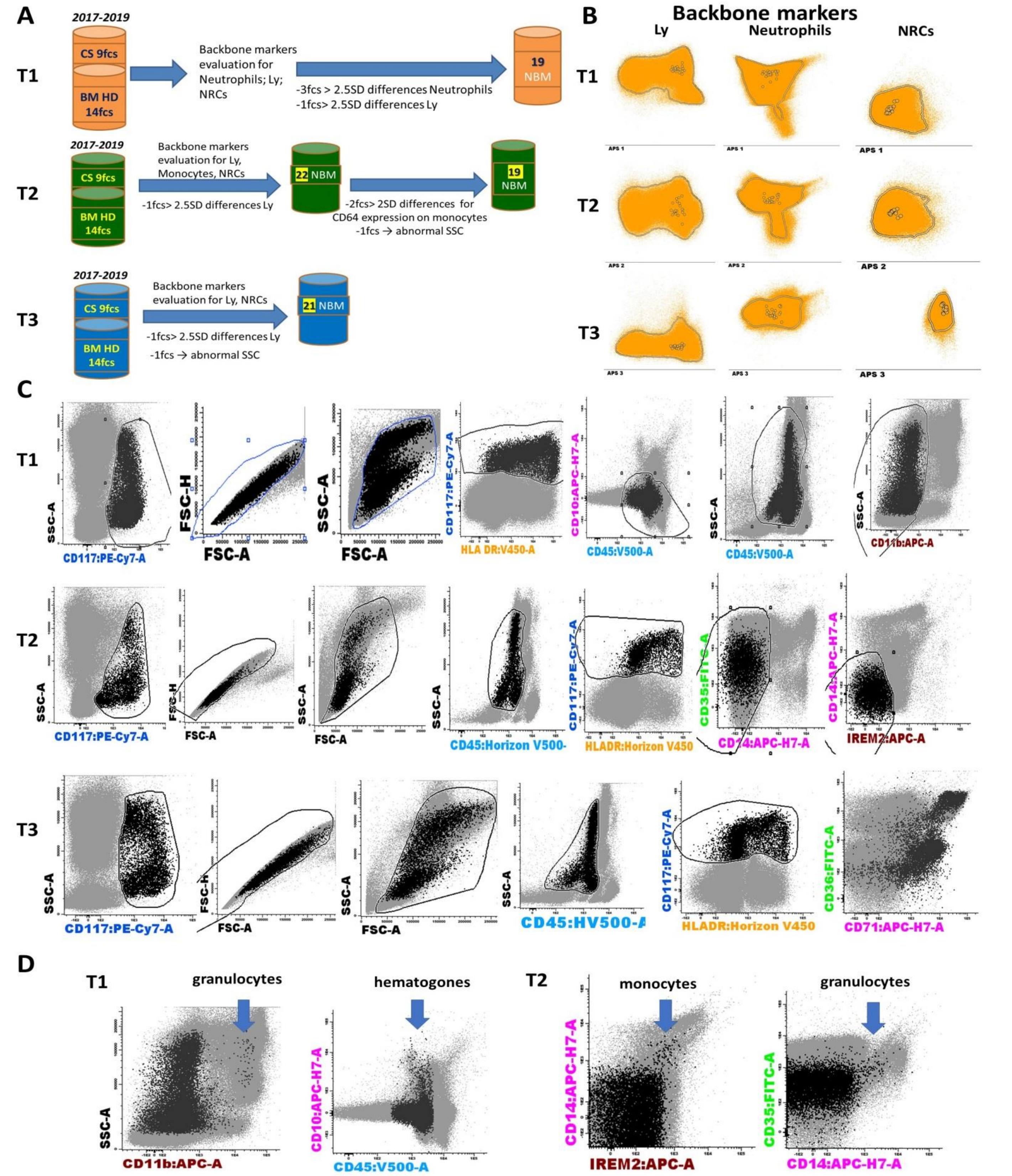

Figure S1. Study design and the strategy of analysis for the identification of normal myHPCs in NBM samples using the Tubes 1-3 of EF AML/MDS panel

(A) Schematic overview of the normal bone marrow (NBM) files included in the databases. FCS files corresponding to the healthy donor (HD) and cardiac surgery (CS) bone marrow (BM) samples stained with antibodies from the EuroFlow (EF) acute myeloid leukemia (AML)/myelodysplastic syndrome (MDS) panel Tube 1 (T1), Tube 2 (T2) and Tube 3 (T3). **(B)** Multidimensional (2 SD principal component-based) views from Tubes 1-3 showing homogenous staining for the backbone markers on the internal control populations (lymphocytes, Ly; neutrophils; and nucleated red cells, NRC) from the NBM files included in the databases. **(C)** Analysis strategy for HPC selection in Tubes 1, 2, and 3 of the EF AML/MDS panel. **(D)** Mature cell populations that may contaminate the blast's gate when the gating strategy is based only on backbone markers. T1, Tube 1; T2, Tube 2.

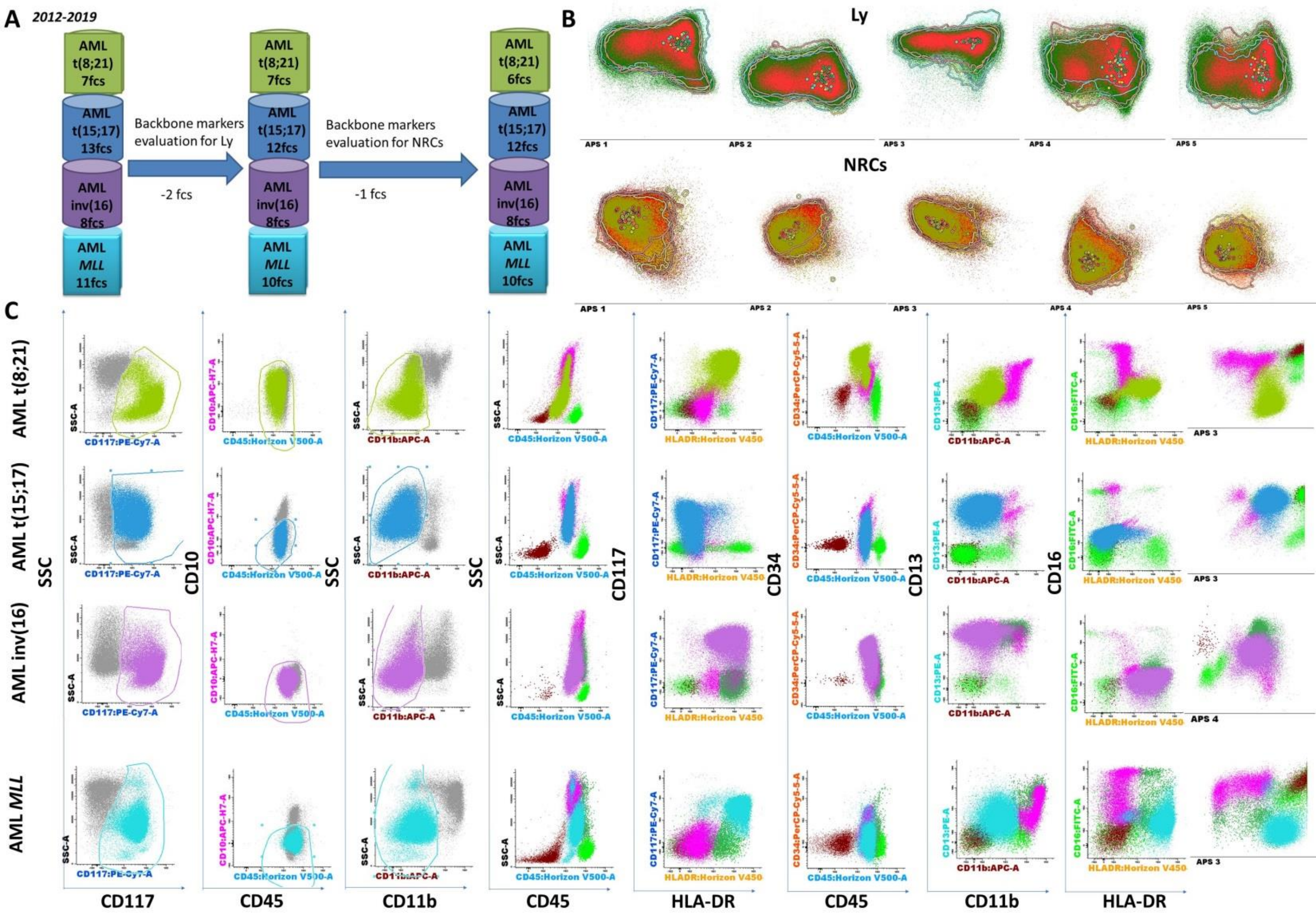

Figure S2. Study design and the strategy of analysis for the identification of AML blasts using Tube 1 of the EF AML/MDS panel

(A) Schematic overview showing the AML files included in the Tube 1 AML database. **(B)** Multidimensional (2 SD principal component-based) views showing homogenous staining for the backbone markers on the internal control populations (lymphocytes, Ly; nucleated red cells, NRC) from the AML FCS files included in the Tube 1 AML database. **(C)** Analysis strategy for selection of AML blast in Tube 1 of the EF AML/MDS panel: t(8;21) AML blasts, yellow-green; t(15;17) AML blasts, light blue; inv(16) AML blasts, violet; *MLL* AML blasts, turquoise; neutrophils, pink; NRC, dark red.; lymphocytes, green.

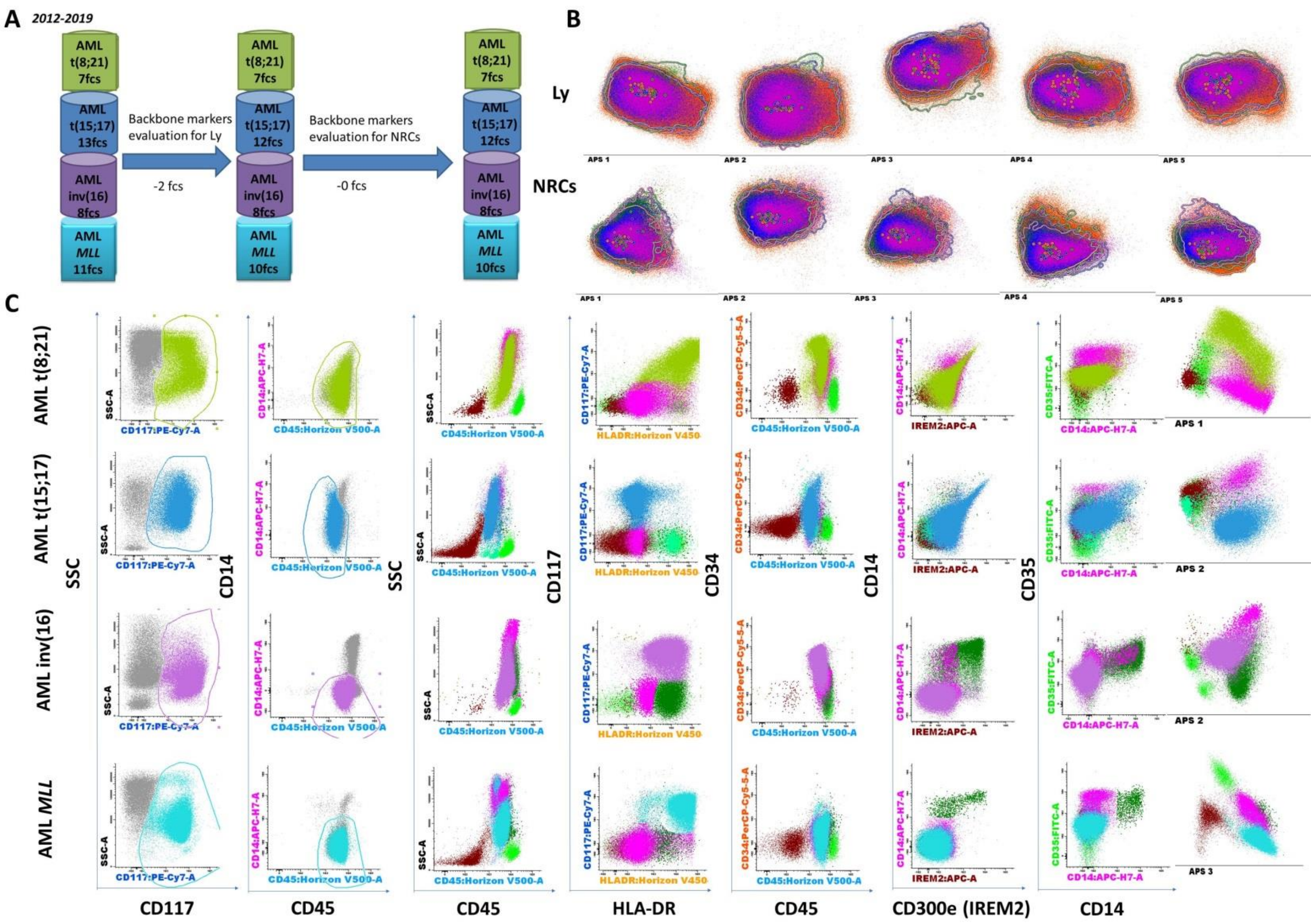

Figure S3. Study design and the strategy of analysis for the identification of AML blasts using Tube 2 of the EF AML/MDS panel

(A) Schematic overview showing the AML files included in the Tube 2 AML database. **(B)** Multidimensional (2 SD principal component-based) views showing homogenous staining for the backbone markers on the internal control populations (lymphocytes, Ly; nucleated red cells, NRC) from the AML FCS files included in the Tube 2 AML databases. **(C)** Analysis strategy for selection of AML blast in Tube 2 of the EF AML/MDS panel: t(8;21) AML blasts, yellow-green; t(15;17) AML blasts, light blue; inv(16) AML blasts, violet; t(9;11) AML blasts, turquoise; neutrophils, pink; NRC, dark red, lymphocytes, green.

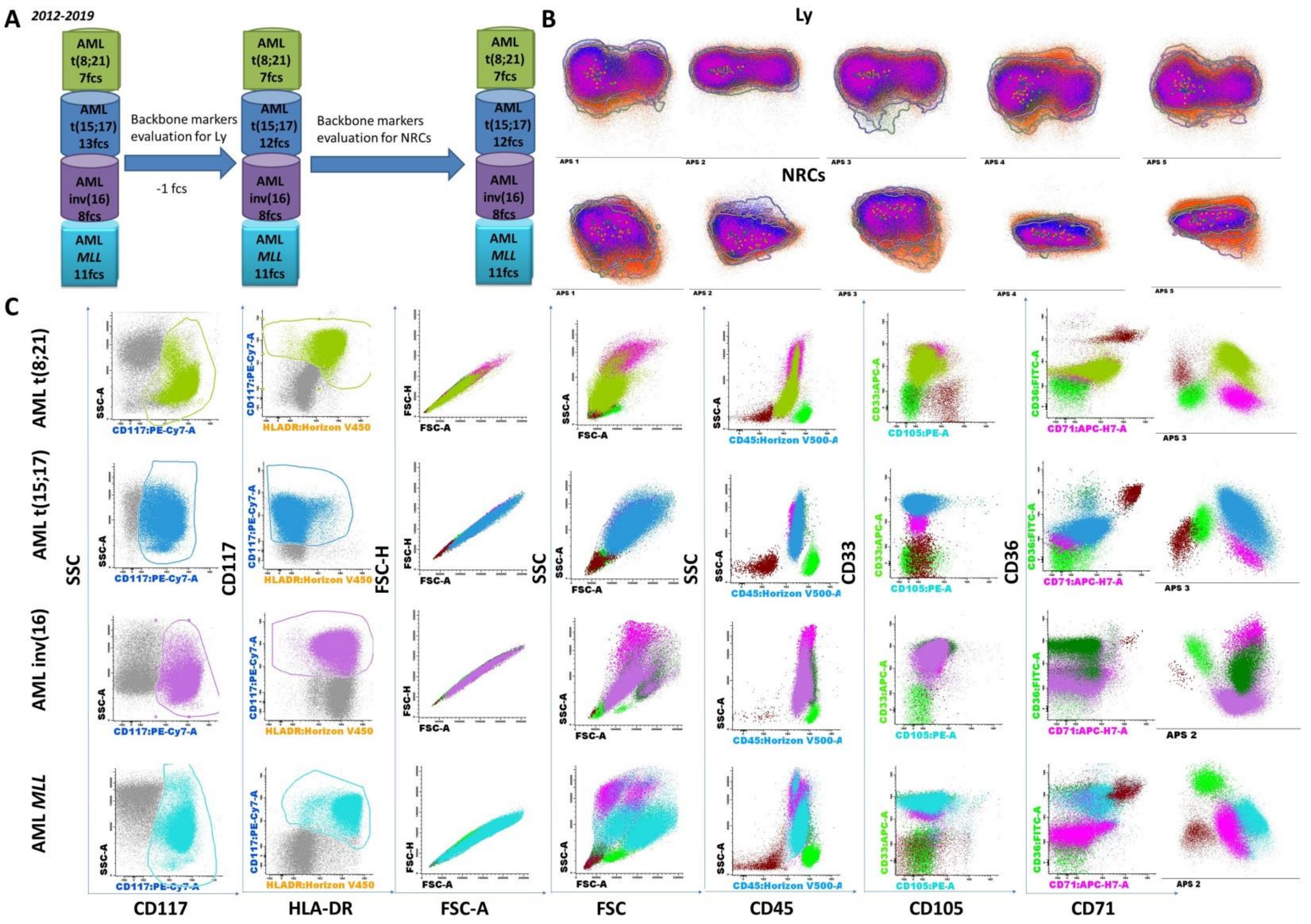

Figure S4. Study design and the strategy of analysis for the identification of AML blasts using Tube 3 of the EF AML/MDS panel

(A) Schematic overview showing the AML files included in the Tube 3 AML database. **(B)** Multidimensional (2 SD principal component-based) views showing homogenous staining for the backbone markers on the internal control populations (lymphocytes, Ly; nucleated red cells, NRC) from the AML FCS files included in the Tube 3 AML databases. **(C)** Analysis strategy for selection of AML blast in the Tube 3 of the EF AML/MDS panel: t(8;21) AML blasts, yellow-green; t(15;17) AML blasts, light blue; inv(16) AML blasts, violet; t(9;11) AML blasts, turquoise; neutrophils, pink; NRC, dark red; lymphocytes, green.

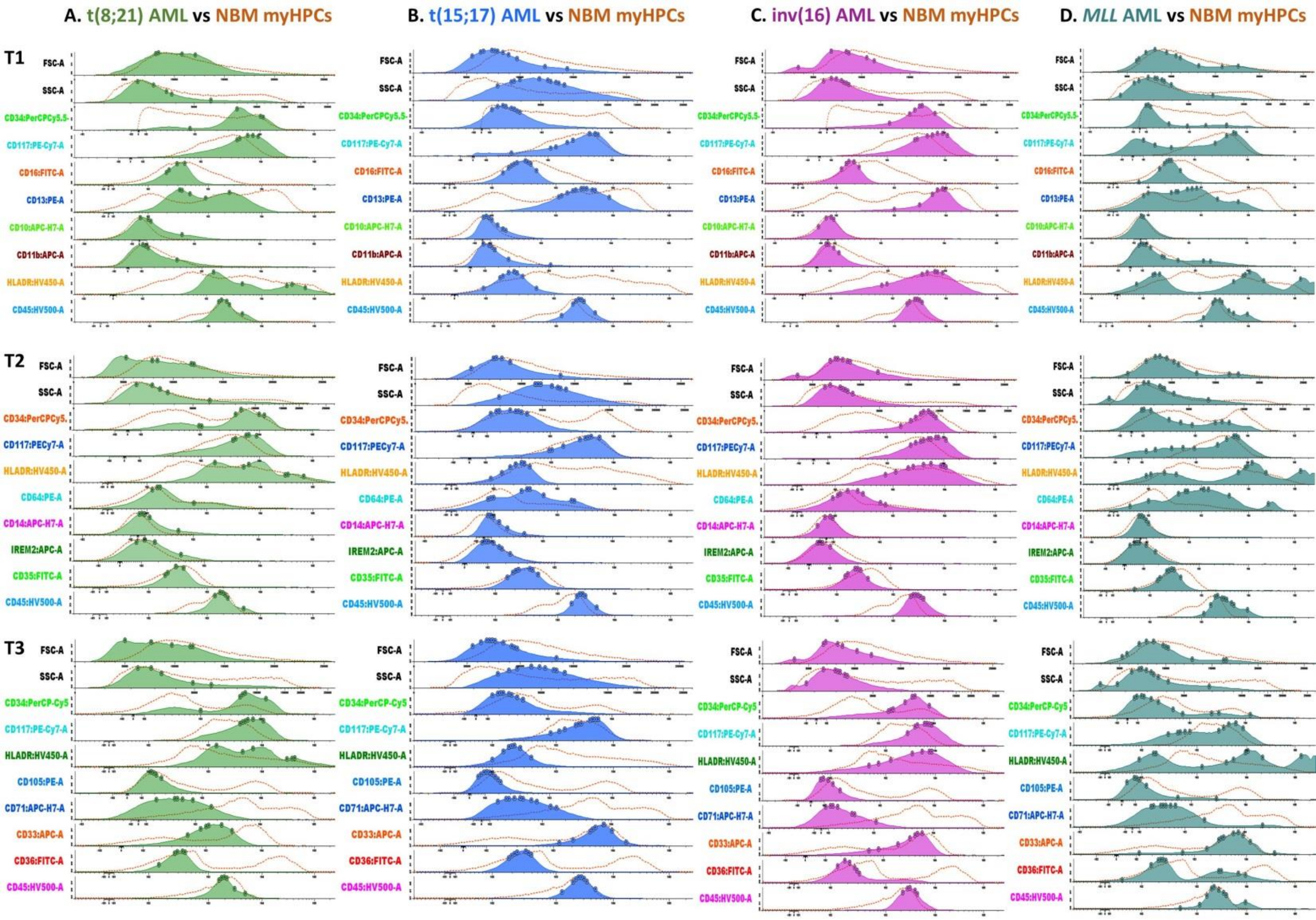

Figure S5. Parameter Band Histograms showing markers expression on AML blasts versus normal myHPCs.

Columns display the phenotype of acute myeloid leukemia (AML) blasts: **(A)** t(8;21) AML blasts, green; **(B)** t(15;17) AML blasts, blue; **(C)** inv(16) AML blasts, violet; **(D)** *MLL* AML blasts, turquoise. Normal myeloid hematopoietic precursor (myHPC) reference images: orange discontinuous line. T1-3 rows correspond to each of Tubes 1–3 of the EuroFlow (EF) AML/myelodysplastic syndrome (MDS) panel. Histograms show the relative expression of each analyzed marker on different groups of AML blasts; circles represent the mean of an individual case; x-axis shows the median intensity of expression (MFI)

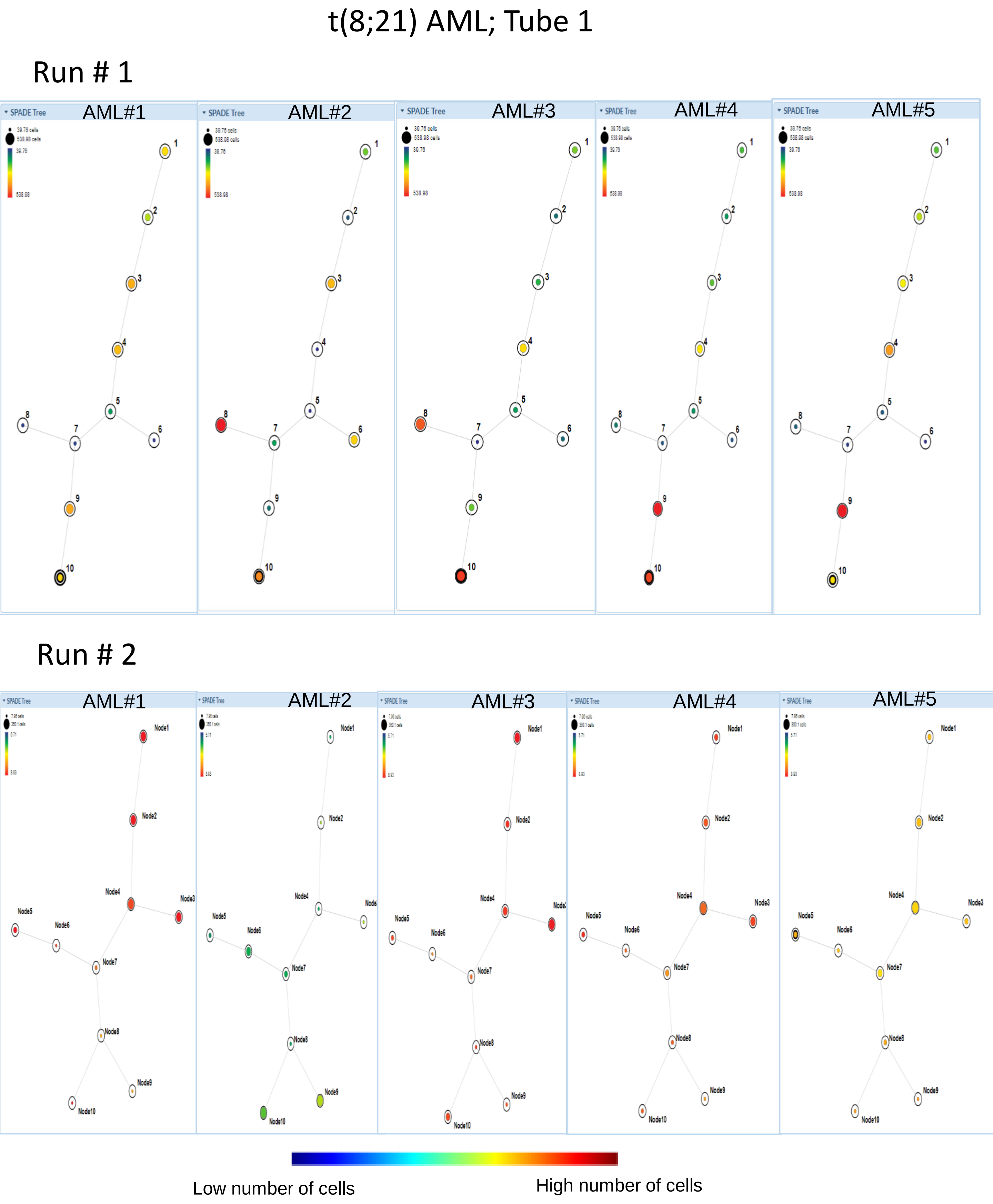

Figure S6. Two SPADE runs of pre-gated AML blasts stained with antibody combinations from Tube 1 EF AML/MDS panel from five t(8;21) AML cases were visually investigated to identify nodes that were potentially different in cellular abundance and in markers expression compared with myHPCs from NBM samples.
In these plots each node comprises a group of phenotypically related cells. The size and the colour of the nodes indicate the abundance and the expression, respectively. Each row represents one of the two SPADE runs and each column represents the SPADE tree obtained for one t(8;21) AML case. Clusters group by AML case are not conserved between runs, resulting in considerable differences between the SPADE tree from the two runs.

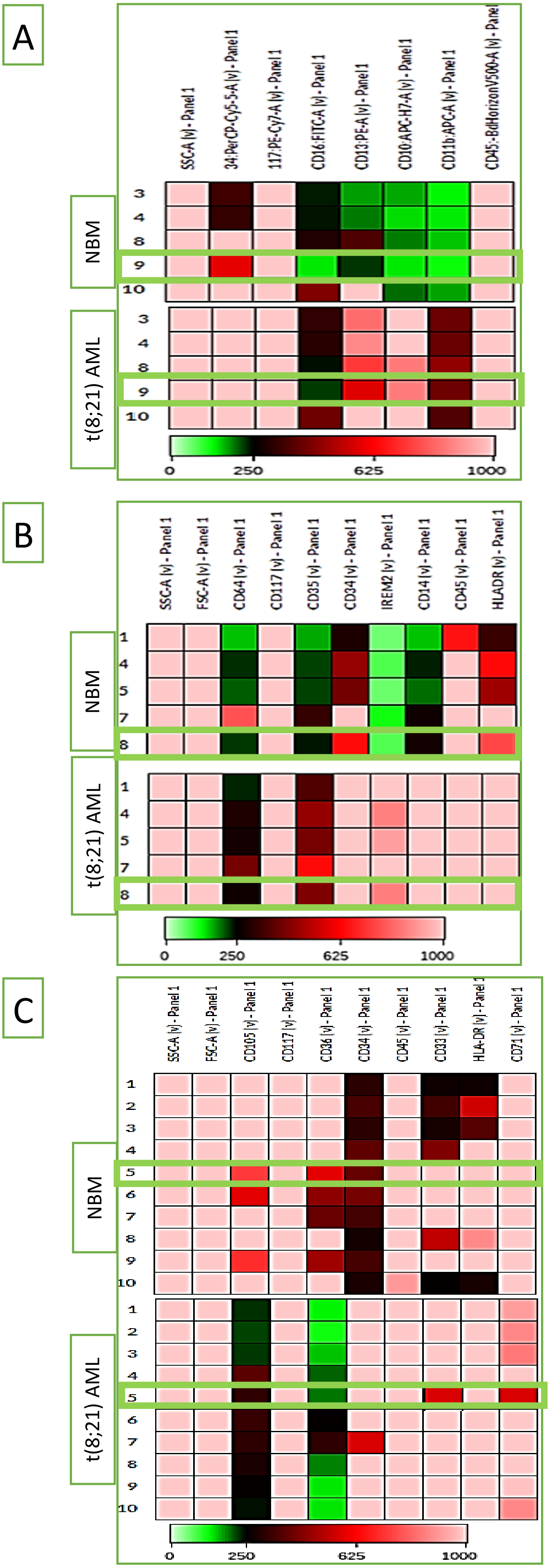

Figure S7. viSNE cluster heatmaps coloured by expression level of indicated markers for AML blasts from a representative t(8;21) AML case (top panel) compared with myHPCs from a NBM sample (bottom panel). Green boxes highlights the nodes containing significantly more events in t(8;21) AML case compared with NBM setting. A. Tube 1 panel EF AML/MDS. B. Tube 2 panel EF AML/MDS. C. Tube 3 panel EF AML/MDS.

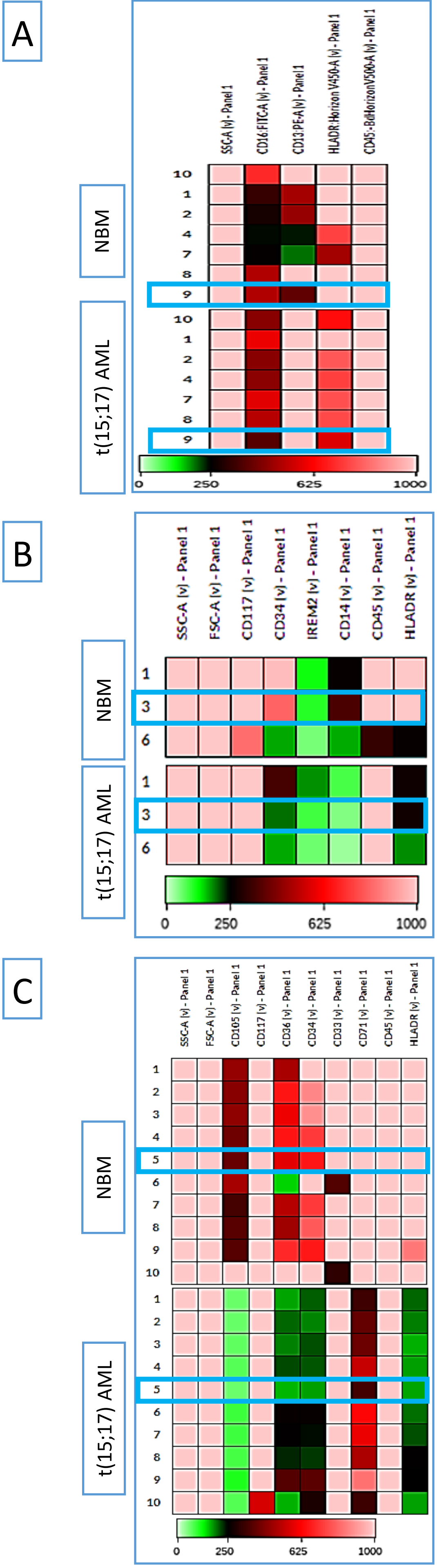

Figure S8. viSNE cluster heatmaps coloured by expression level of indicated markers for AML blasts from a representative t(15;17) AML case (top panel) compared with myHPCs from a NBM sample (bottom panel). Green boxes highlights the nodes containing significantly more events in t(15;17) AML case compared with NBM setting. A. Tube 1 panel EF AML/MDS. B. Tube 2 panel EF AML/MDS. C. Tube 3 panel EF AML/MDS.

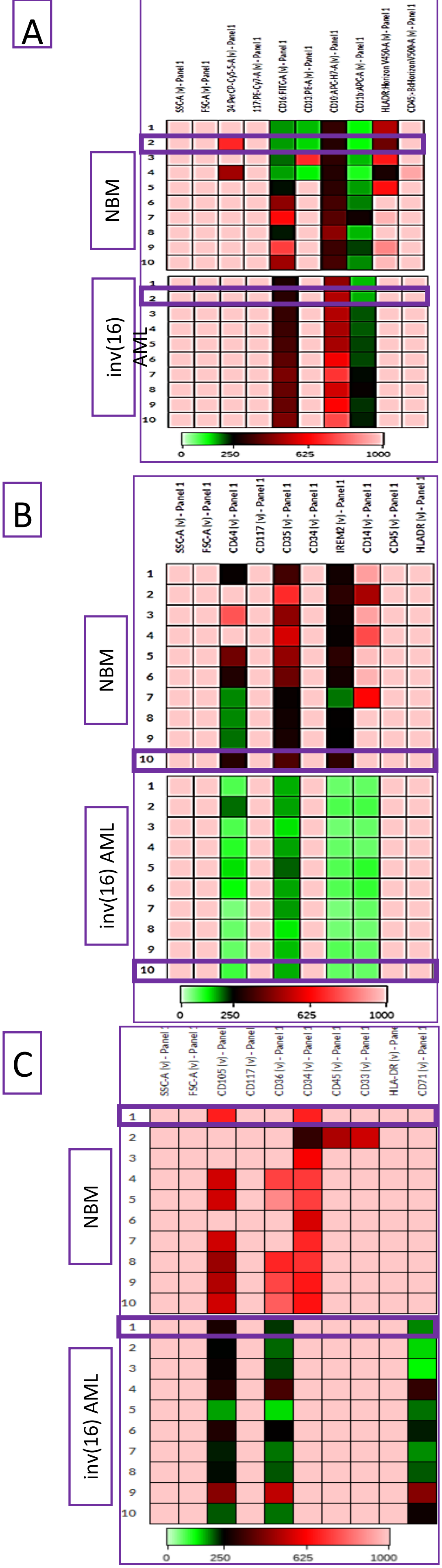

Figure S9. viSNE cluster heatmaps coloured by expression level of indicated markers for AML blasts from a representative inv(16) AML case (top panel) compared with myHPCs from a NBM sample (bottom panel). Violet boxes highlights the nodes containing significantly more events in inv(16) AML case compared with NBM setting. A. Tube 1 panel EF AML/MDS. B. Tube 2 panel EF AML/MDS. C. Tube 3 panel EF AML/MDS.

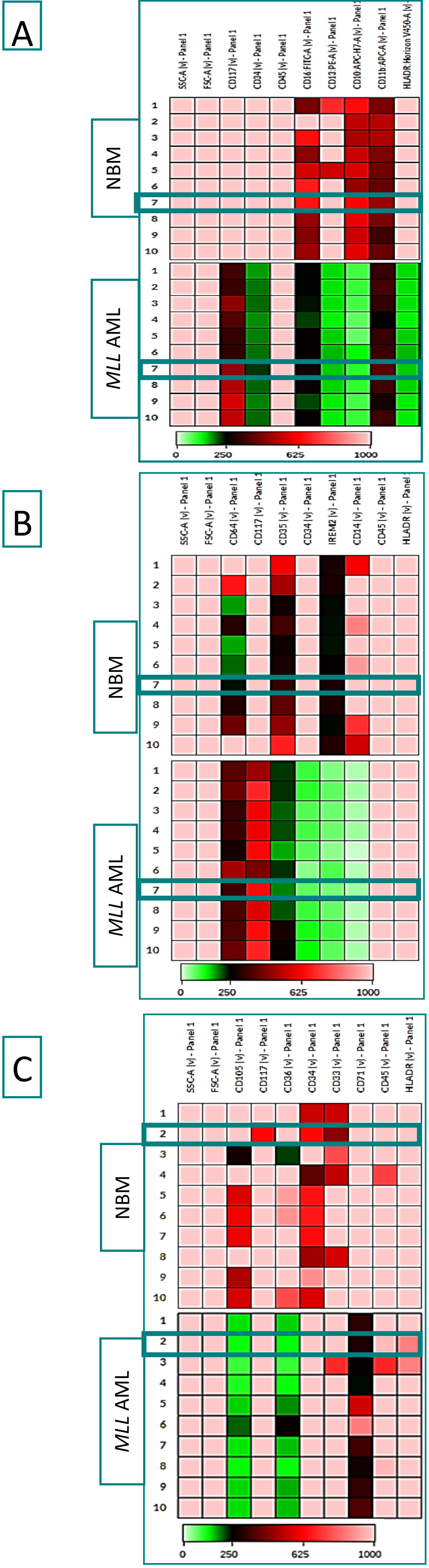

Figure S10. viSNE cluster heatmaps coloured by expression level of indicated markers for AML blasts from a representative *MLL* AML case (top panel) compared with myHPCs from a NBM sample (bottom panel). Turquoise boxes highlights the nodes containing significantly more events in *MLL* AML case compared with NBM setting. A. Tube 1 panel EF AML/MDS. B. Tube 2 panel EF AML/MDS. C. Tube 3 panel EF AML/MDS.
